# Thirteen-Year Trends in Dietary Patterns among Japanese Adults in the National Health and Nutrition Survey 2003–2015: Continuous Westernization of the Japanese Diet

**DOI:** 10.3390/nu10080994

**Published:** 2018-07-30

**Authors:** Kentaro Murakami, M. Barbara E. Livingstone, Satoshi Sasaki

**Affiliations:** 1 Department of Social and Preventive Epidemiology, School of Public Health, University of Tokyo, Tokyo 113-0033, Japan; stssasak@m.u-tokyo.ac.jp; 2 Nutrition Innovation Centre for Food and Health (NICHE), School of Biomedical Sciences, Ulster University, Coleraine BT52 1SA, UK; mbe.livingstone@ulster.ac.uk

**Keywords:** dietary patterns, food consumption patterns, principal component analysis, trend analysis, national survey

## Abstract

We examined 13-year trends in dietary patterns, using data from the National Health and Nutrition Survey, Japan 2003–2015. In repeated, independent cross-sectional studies, dietary intake was assessed with a one-day weighed dietary record in 88,527 Japanese adults aged ≥20 years. Using principal component analysis based on the daily consumption of 31 food groups, we identified three dietary patterns, the “plant food and fish”, “bread and dairy”, and “animal food and oil” patterns. In the whole sample, the “plant food and fish” pattern score decreased while the “bread and dairy” and “animal food and oil” pattern scores increased. The decreasing trends in the “plant food and fish” pattern were observed in all subgroups considered. The increasing trends in the “bread and dairy” pattern were similar across sexes and by current smoking status. However, in terms of age, occupation, and weight status, the increasing trends were only evident in particular subgroups (i.e., age 50–64 and ≥65 years; security/transportation/labor occupation and nonworker; and normal weight and overweight participants). For the “animal food and oil” pattern, the increasing trends were observed in all subgroups, except for the youngest age group (20–34 years old). This study suggests continuous Westernization of the Japanese diet.

## 1. Introduction

Understanding trends in dietary habits is critical to priority setting and policy making aimed at improving diets and reducing diet-related illness [1]. Since the potential synergistic effects of foods and nutrients cannot be investigated using the traditional single food and nutrient approach, nutritional epidemiology has recently made a shift in emphasis to the assessment of dietary patterns [2]. Nevertheless, we are aware of no trend analysis in dietary patterns based on nationally representative datasets in Asia. Were it available, this kind of information would be useful in developing country-specific nutrition policies and food-based dietary guidelines.

The two main approaches to derive dietary patterns are the a priori (hypothesis-derived) and a posteriori (data-derived) approaches. The latter is suitable for deriving a representative picture of the diet in a specific population, and has been used in many studies [3,4,5,6,7,8,9,10,11,12,13,14,15,16]. The most commonly used method for these analyses is principal component analysis (PCA). This data reduction method is based on the correlation metrics of the original variables, which is used to produce a set of values for linearly uncorrelated variables (patterns).

Partly as a result of the low prevalence of coronary artery disease and long life expectancy of the Japanese population, dietary habits in Japan have long been of interest to researchers from other countries [17,18]. Recent Japanese diets typically include high intakes of refined grains (mainly white rice), seaweeds, vegetables, fish, soybean products, and green tea, as well as low intakes of whole grains, processed meat, nuts, and soft drinks [19,20]. While a number of Japanese studies have utilized PCA to determine dietary patterns [21,22,23,24,25,26,27,28,29,30], recent secular trends in dietary patterns remain unknown. Data from food balance sheets from 1960 to 2005 suggest that the Japanese diet has been undergoing Westernization: during this period, the per-capita supply of meat/poultry and fats/oils markedly increased, the rice supply sharply decreased, and the supply of vegetables, fruits, and fish/shellfish remained relatively stable [31]. Here, to investigate whether the Westernization of the Japanese diet is still continuing, we conducted a trend analysis of PCA-derived dietary patterns in free-living Japanese adults consuming a self-selected diet using food intake data from the National Health and Nutrition Survey, Japan (NHNSJ) 2003–2015.

## 2. Materials and Methods

### 2.1. Data Source and Analytic Sample

**Figure 1:**
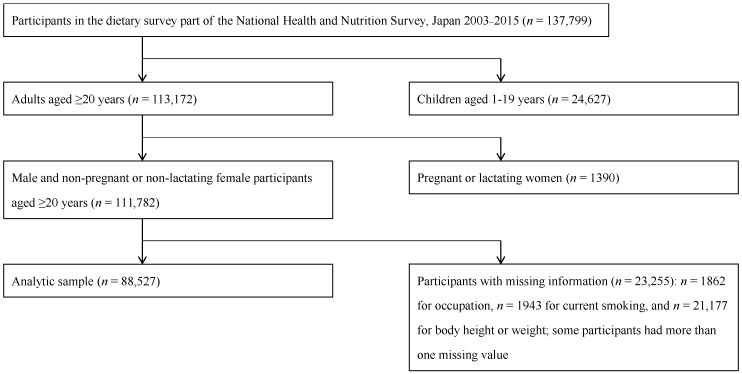
Flow diagram of the study participants.

Details of the NHNSJ have been published elsewhere [20,32]. Briefly, the NHNSJ is an annual national nutrition survey that has been conducted since 1945 by local public health centers under the supervision of the Ministry of Health, Labour and Welfare. The present analysis was based on data from the NHNSJ 2003–2015 owing to the availability of individual-level data on food group intake. A stratified two-stage cluster sampling was used to obtain a representative sample of the non-institutionalized Japanese population aged ≥1 year, with the exception of the 2012 survey, in which a stratified single-stage cluster sampling was used to expand the sample size. The survey was conducted in November in each year, except for the 2012 survey (i.e., late November to early December). All regions in Japan were represented at each survey. The number of households taking part in the survey ranged from 3412 (in 2011) to 12,750 (in 2012), and had an overall response rate of around 50%. While there is a theoretical possibility of duplication of participants in the NHNSJ over the years, this should be practically negligible considering the huge number of households in Japan (>50 million). In total, 137,799 individuals participated in the dietary survey part of NHNSJ 2003–2015. Of these, the number of participants aged ≥20 years, was 113,172. We excluded lactating or pregnant women (*n* = 1390) because these were presumably not following their usual diet. After further excluding individuals with missing information on the variables of interest (*n* = 23,255), the final analytic sample comprised 88,527 adults aged ≥20 years, (Figure 1). Not only because there were only a few extreme reporters of energy intake (i.e., *n* = 132 for <500 kcal/day and 38 for >5000 kcal/day) but also because further exclusion of these participants did not alter the findings of the present study (data not shown), these participants were included in the analysis.

The NHNSJ was conducted according to the guidelines laid down in the Declaration of Helsinki, and verbal informed consent was obtained from each participant. Under the Statistics Act, the Ministry of Health, Labour and Welfare anonymized individual-level data collected from the NHNSJ and provided the first author (Kentaro Murakami) with the datasets for this study. In accordance with the Ethical Guidelines of Epidemiological Research established by the Ministry of Education, Culture, Sports, Science and Technology and the Ministry of Health, Labour and Welfare, an institutional review board approval was not required for this analysis.

### 2.2. Dietary Assessment

Dietary intake data were obtained using a one-day weighed household dietary record, as detailed previously [32,33,34]. The dietary record therefore included data on all members of the household. In brief, the participant and the main record keeper were supplied with a diary for recording and given written and verbal instructions in the home from trained fieldworkers (registered dietitians) on how the diary should be maintained. The main record keeper was asked to weigh and record all food and beverage items (except for drinking water) consumed by the household members during the day of recording. The equipment used to weigh foods and beverages (which is usually already present in Japanese households) was not provided mainly due to funding limitations. Typically, the main record keeper was asked to weigh ingredients used during food preparation. Where household members shared food items from the same dish, the record keeper was requested to record the approximate proportions of food taken by each member to allow the dietary intake of each individual to be estimated. Where weighing was not possible (e.g., eating out), the record keeper was asked to record as much detail as possible, including portion size and leftovers. To ensure maximum participation, the recording day could be freely selected by the household from any day, excluding Sundays, national holidays, and days with special events, such as wedding parties and funerals. Although the survey did not formally collect information on the identity of the main record keeper, the survey assumed that recording was undertaken by the main household cook, who in Japan is usually a woman. Within a short time after recording (usually the next weekday), trained fieldworkers went to each household to collect the diary, and also to confirm the completeness of food recording. Additional information was added were necessary.

In accordance with a study manual of the NHNSJ, trained fieldworkers converted the estimates of portion sizes into weights (for only food items recorded using household measures), and coded all individual food items on the basis of the Standard Tables of Food Composition in Japan [35]. The collected dietary records were then checked at the local center, where trained fieldworkers input the dietary intake data using software custom-developed for the NHNSJ. These were then compiled by trained investigators at the central office to produce the overall dietary dataset.

The value of this household dietary record in estimating individual-level dietary intake in Japanese has been examined [36]. Briefly, dietary intakes among young women (about 20 years old of age) estimated using this one-day household dietary record by mothers (mean age: 49 years) were compared with those estimated using a one-day weighed dietary record that was independently conducted by the young women themselves (*n* = 32). Mean intake differences between the methods were 6.2% for energy, 5.7% for protein, 6.7% for fat, and 6.3% for carbohydrate, whereas Pearson correlation coefficients were 0.90 for energy, 0.89 for protein, 0.91 for fat, and 0.90 for carbohydrate. Further, previous analyses using the NHNSJ 2012 showed mean values for the ratio of energy intake to estimated energy requirement of 1.04 for children [37] and 0.98 for adults [38].

### 2.3. Identification of Dietary Patterns

Information on 98 food items consumed by the participants was available from the individual-level dataset of NHNSJ 2003–2015. These are subsidiary food groups based on categories predefined by NHNSJ; information on intake of more aggregated food items is not available. Before dietary pattern analysis, these 98 food items were recoded into 31 food groups based on culinary usage of the food to better reflect actual food consumption patterns (Table S1). To derive dietary patterns, PCA was performed based on intakes of the 31 food groups expressed as amount per day, using the PROC FACTOR procedure in SAS (version 9.4, SAS Institute Inc., Cary, NC, USA). The derived factors are linear combinations of the included variables which explain as much of the variation in the original variables as possible. The factors were rotated by orthogonal transformation (varimax rotation) to provide a simpler structure having greater interpretability. The number of retained factors was determined by evaluating the scree plot and the combination of food groups for the identified factors [21]. The proportion of variance explained by the respective factors was determined by dividing the sum of squares of the respective factor loadings by the number of variables (i.e., food groups). Factor loadings are correlation coefficients between individual food groups and dietary patterns. Food groups having absolute factor loadings of ≥0.30 were determined to have contributed to a factor [10,12,13,39]. Dietary patterns were described based mainly on these food groups, and factor scores for each participant and for each dietary pattern were obtained by adding the standardized intake of each of the 31 food groups weighted by the factor loading for each pattern. These scores represent standardized variables with a mean of 0 and a standard deviation of 1.

### 2.4. Assessment of Basic Characteristics

In the NHNSJ 2012–2015, body height (to the nearest 0.1 cm) and weight (to the nearest 0.1 kg) were measured in 67% of the participants by trained fieldworkers using standardized procedures. For the remaining participants (33% in the NHNSJ 2012–2015), height and weight were either measured by other household members at home or self-reported. In the NHNSJ 2003–2011, height and weight measurement was similarly obtained although information on the measurement procedure was not available. Body mass index (BMI; kg/m^2^) was calculated as weight (kg) divided by the squared height (m^2^). Weight status was defined based on BMI according to World Health Organization recommendations as follows [40]: underweight (<18.5 kg/m^2^), normal weight (≥18.5 to <25 kg/m^2^), and overweight (including obese; ≥25 kg/m^2^). Using a self-administered questionnaire, information on occupation (professional/manager, sales/service/clerical, security/transportation/labor, or nonworker) and current smoking (no or yes) was collected. Age was categorized using 15-year age bands as follows: 20–34, 35–49, 50–64, and ≥65 years.

### 2.5. Statistical Analysis

All statistical analyses were performed using SAS statistical software (version 9.4, SAS Institute Inc., Cary, NC, USA). Differences in basic characteristics and food group intakes between the participants included in the analysis and those excluded from the analysis were examined based on the independent *t*-test for continuous variables and the chi-square test for categorical variables. Trends in the characteristics of the participants were examined by linear regression for continuous variables (with modeling of the survey year as a continuous variable) and by the Mantel-Haenszel chi-square test for categorical variables.

Trends in dietary pattern scores were evaluated by linear regression, using dietary pattern score as the dependent variable and survey year as the independent variable. Mean and standard error scores of each dietary pattern were calculated according to survey year. To calculate *p* values for trend, a linear trend test was used with the survey year used as a continuous variable in linear regression. Per-year change in dietary pattern score (i.e., regression coefficient) was also calculated using linear regression. Adjustment was made for sex, age category, occupation, weight status, and current smoking. Trend analysis was also conducted after participants were stratified by these variables. Interactions between these variables and survey year were examined by adding the product term of the time and variable of stratification into the linear regression model. Because the 2012 survey differed from the other surveys in terms of sampling design and sample size, these analyses were repeated after excluding the 2012 data. Because of the large sample size and number of statistical tests performed, significance was considered for two-tailed tests at *p* < 0.0001.

## 3. Results

### 3.1. Characteristics of the Analytic Sample

This analysis included 88,527 male and nonlactating and nonpregnant female participants aged ≥20 years, who differed from those excluded from the analysis because of missing information. The excluded participants were more likely to be in the 2012 survey, male, younger, in the occupation categories of professional/manager or sales/service/clerical, have a smaller BMI, and be current smokers (Table S2). Differences between the included and excluded participants were also observed in the intakes of almost all food groups (Table S3).

Characteristics of the analytic sample by survey year are shown in Table 1. Over the study period, the percentages of older participants, non-working participants, and nonsmokers increased while the percentages of younger participants, those categorized in the security/transportation/labor occupation, and smokers decreased.

### 3.2. Dietary Patterns

The PCA identified three dietary patterns (Table 2). Factor 1, characterized by high intakes of green and yellow and other vegetables, fruits, pulses, potatoes, mushrooms, seaweeds, pickled vegetables, rice, fish, sugar, salt-based seasonings, and tea, was labelled the “plant food and fish” pattern. Factor 2, characterized by high intakes of bread, dairy products, fruits, and sugar and low intake of rice, was labelled the “bread and dairy” pattern. Factor 3, characterized by high intakes of red and processed meat, eggs, vegetable oil, and other vegetables, was labelled the “animal food and oil” pattern. Overall, the three dietary patterns explained 18.63% of the variance in food group intake.

### 3.3. Trends in Dietary Patterns

**Figure 2:**
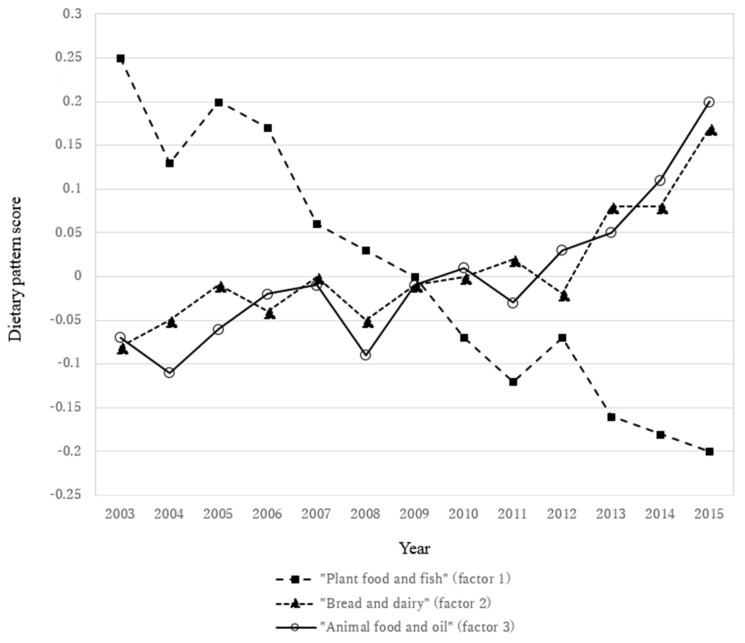
Thirteen-year trends in dietary pattern scores in adults aged ≥20 years in the National Health and Nutrition Survey, Japan 2003–2015. Values are means adjusted for sex, age category, occupation, weight status, and current smoking. The dietary pattern score represents standardized variables with mean 0 and standard deviation 1. Negative scores indicate low adherence to the dietary pattern, and positive scores indicate high adherence. The naming of the dietary patterns was as follows: “plant food and fish” (factor 1), “bread and dairy” (factor 2), and “animal food and oil” (factor 3). Sizes of the study population were as follows: *n* = 7062 for 2003, *n* = 5675 for 2004, *n* = 5469 for 2005, *n* = 6062 for 2006, *n* = 5954 for 2007, *n* = 6198 for 2008, *n* = 6047 for 2009, *n* = 5581 for 2010, *n* = 5197 for 2011, *n* = 19,717 for 2012, *n* = 5393 for 2013, *n* = 5298 for 2014, and *n* = 4874 for 2015. The trends in all dietary patterns were significant (*p* for trend < 0.0001).

The 13-year trends in the three dietary patterns are shown in Figure 2. Negative scores express low adherence to dietary pattern while positive scores show high adherence. After adjustment for sex, age category, occupation, weight status, and current smoking, the “plant food and fish” pattern score decreased during the study period while the “bread and dairy” and “animal food and oil” pattern scores increased. These dietary pattern trends were generally well reflected by the trends in food group intakes (Table S4).

**Figure 3:**
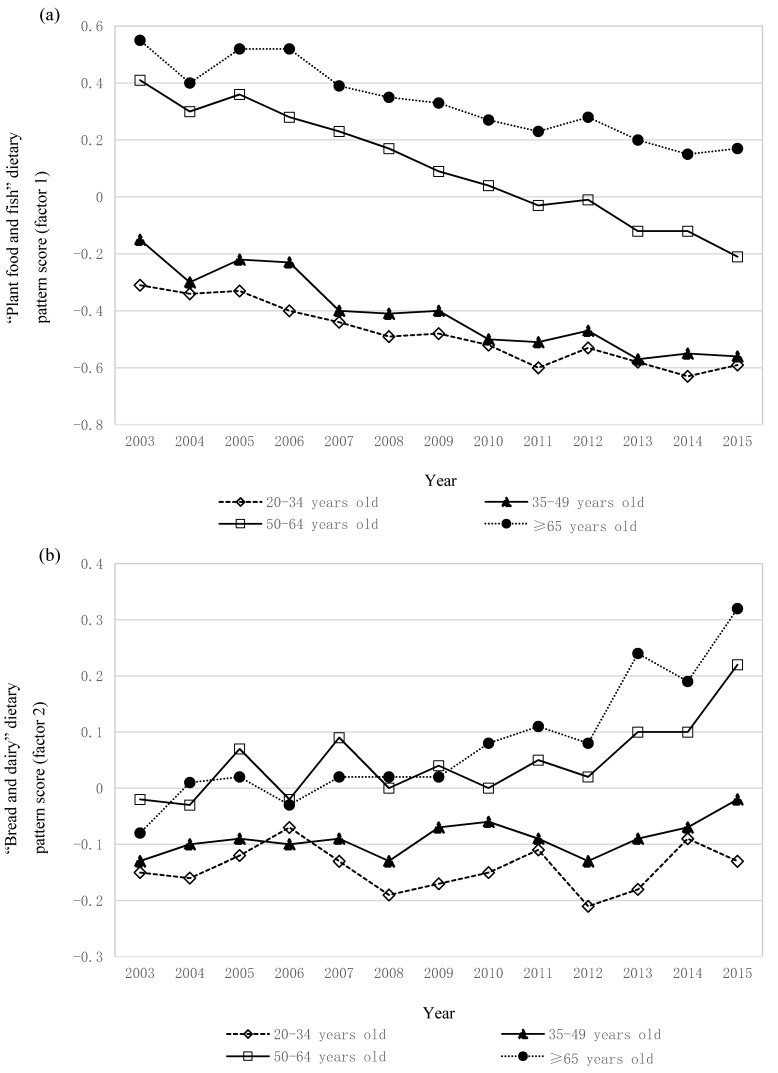

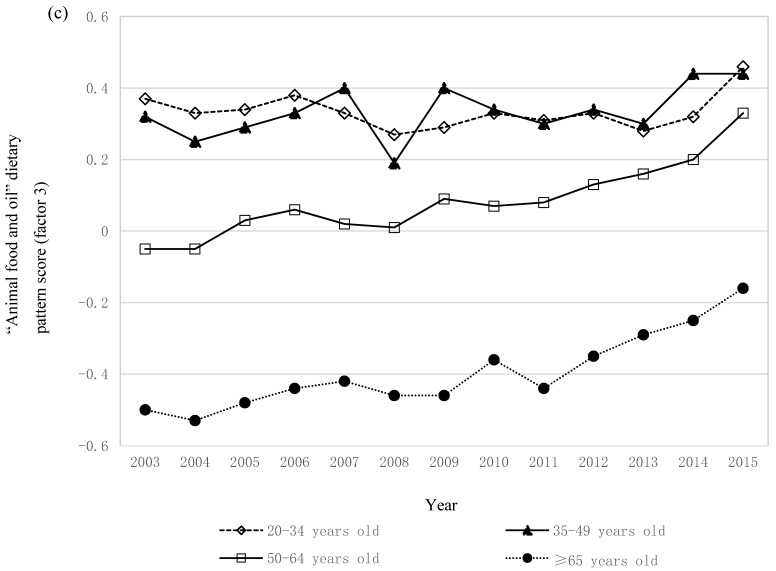
Thirteen-year trends in the (**a**) “plant food and fish”, (**b**) “bread and dairy”, and (**c**) “animal food and oil” dietary pattern scores stratified by age category. Values are means adjusted for sex, occupation, weight status, and current smoking. The dietary pattern score represents standardized variables with mean 0 and standard deviation 1. Negative scores indicate low adherence to the dietary pattern, and positive scores indicate high adherence. Data were derived from the National Health and Nutrition Survey 2003–2015. Sizes of the study population were 10,983, 19,727, 25,732, and 32,085 for participants aged 20–34, 35–49, 50–64, and ≥65 years, respectively. The trends in all dietary patterns were significant (*p* for trend < 0.0001), except for “bread and dairy” in participants aged 20–34 years (*p* for trend = 0.19) and 35–49 years (*p* for trend = 0.11) and “animal food and oil” in those aged 20–34 years (*p* for trend = 0.68).

Similar to the analysis based on the whole sample, the decreasing trends in the “plant food and fish” pattern score were observed in all subgroups considered (Table S5), including age categories (Figure 3a). In addition, the increasing trends in the “bread and dairy” pattern score were similar between sexes and current smoking (Table S6). However, in terms of age category (Figure 3b), the increasing trends were only evident in older participants (i.e., those aged 50–64 and ≥65 years). Additionally, the increasing trends were observed in the occupation categories of security/transportation/labor and nonworker, but not in those of professional/manager or sales/service/clerical. For weight status, the increasing trends were observed in normal weight and overweight participants but not in underweight participants. For the “animal food and oil” pattern score (Table S7), the increasing trends were observed in all subgroups considered, except for the 20–34 years old age group (Figure 3c). The dietary pattern analysis (Table S8) and all the trend analyses repeated after excluding the 2012 survey data (because of difference in sampling design and sample size) provided similar findings in terms of the “plant food and fish”, “bread and dairy”, and “animal food and oil” patterns (Tables S9–S11, respectively).

## 4. Discussion

To our knowledge, this is the first study to investigate trends in PCA-derived dietary patterns using a nationally representative dataset. During the period 2003–2015, the “plant food and fish” pattern score decreased while the “bread and dairy” and “animal food and oil” pattern scores increased in Japan, particularly among older age groups, suggesting the continuous Westernization of the Japanese diet.

In spite of large differences in dietary habits among countries, studies from a range of countries have consistently identified two major dietary patterns [5,6,7,8,9,10,11,12,13,14,15,16], a ‘healthy/prudent’ pattern, characterized by a high intake of fruit, vegetables, whole grains, fish and seafood, legumes, olive oil, nuts, seeds, and low-fat dairy products; and a ‘Western/unhealthy’ pattern, characterized by a high intake of meat (mainly processed), refined grains, sweets, and soft drinks. The “plant food and fish” dietary pattern revealed in the present study had certain characteristics consistent with the former, whereas the “animal food and oil” dietary pattern had certain characteristics in common with the latter. Further to these two patterns, previous Japanese studies similarly identified a third pattern (i.e., the “bread and dairy” pattern) [21,22,23,24,25], which may be considered neither healthy nor unhealthy owing to an extremely low intake of whole grain foods (including bread) in Japan [41,42] and potential health benefits of dairy products [43]. The present study, based on data from national dietary surveys, thus confirms these major patterns in the Japanese diet.

Nevertheless, one important feature is the appearance of rice in the somewhat healthy pattern (i.e., the “plant food and fish” pattern). This has not been previously observed [22,23,24,25], albeit that rice contributed to another dietary pattern in combination with miso soup in some studies [21,26,27,28,29,30]. The reason for this is unknown, but may be due to the use of dietary datasets collected over a longer period of time. Because rice is the top contributor to the intake of energy and many nutrients [20], further investigation is warranted. Additionally, it should be noted that the “plant food and fish” pattern had a high positive loading of salt-based seasonings, the top contributor to sodium intake in Japanese diet [44,45]. Thus, continuous efforts for reducing seasonings should be considered independent of dietary patterns.

This 13-year (2003–2015) trend analysis revealed that the “plant food and fish” pattern score decreased while the “bread and dairy” and “animal food and oil” pattern scores increased. This study may be considered evidence for the continuous Westernization of diets in Japan more broadly identified during the period 1960–2005 by food balance sheets [31]. While the decreasing trend in the “plant food and fish” pattern was observed in all subgroups considered, the increasing trends in the “bread and dairy” and “animal food and oil” patterns were more evident in older rather than younger participants. The reason is unknown, but one possibility is that younger participants had already experienced a shift to Westernization before the study period whereas older participants did so during the study period. This might also explain the lack of increasing trends in the “bread and dairy” pattern in those categorized into professional/manager and sales/service/clerical occupations (mean age: 48.3 and 47.4 years, respectively), given that they were younger than those categorized into security/transportation/labor occupation and nonworkers (mean age: 53.3 and 66.0 years, respectively).

While this study generally showed a change in dietary pattern toward somewhat unhealthy eating, a limited number of similar trend analyses have shown the opposite findings. In a representative sample in Geneva, Switzerland, the score for the “fish and vegetables” pattern increased while those for the “meat and chips” and “chocolate and sweets” patterns decreased during the period 1993–2013 [46]. Moreover, in nationally representative US surveys conducted from 1999 to 2012, the summary score of diet quality showed significant improvement [1]. While it is not possible to determine which countries or populations have better diet quality based on different measures of dietary patterns, this study may suggest the necessity for monitoring of dietary patterns in Japan to improve dietary intake at the population level.

Several limitations of this study are acknowledged. First, although the NHNSJ aims for a nationally representative sample of the noninstitutionalized population of Japan, only about 50% of sampled households participated. Furthermore, information on the basic characteristics of households that refused to participate was not available, and the exact response rate at the individual level was not determined [20]. Additionally, as mentioned above, differences in basic characteristics and food group intakes were observed between the participants included in the analysis and those excluded from the analysis. Thus, while the datasets used should reflect a highly representative picture of dietary patterns in Japan, the possibility of selection bias cannot be excluded.

Self-reported dietary assessment is subject to random and systematic measurement errors [47,48]. Considering that dietary intake varies day-to-day among free-living individuals, the dietary patterns revealed in this study from one-day weighed household dietary records do not likely represent the usual patterns of individual respondents. Nevertheless, they are able to provide accurate population averages. Further, the day of the week on which the dietary assessment was done was not proportionately selected, and in accordance with the survey protocol, Sundays were excluded. This likely also produced some bias in the assessment of average dietary patterns. We do not know which day was selected for dietary recording [20]. In addition, given that the survey was performed within a single month (November), the possibility of seasonal variation was also not considered. This might also have introduced bias into the assessment of average dietary patterns, considering seasonal differences in food intake among Japanese (e.g., higher fruit and lower vegetable intake in fall compared with other seasons) [49]. Furthermore, self-reported dietary assessment methods suffer from the problems of misreporting of dietary intake, particularly among overweight and obese individuals [47,48], albeit that repeated analysis in our study after further adjustment for energy intake produced similar results. Most importantly, the use of this household dietary record to evaluate dietary intake in individuals has been validated among young women, but not in women of other age groups or men [36], and its actual validity in the assessment of dietary patterns is unknown. Assessment of usual dietary patterns with several days of dietary assessment would have been preferable, especially if all seasons and days of the week were included. Use of a validated dietary assessment questionnaire would also have been preferable. The NHNSJ should consider the feasibility of these suggestions.

The methods used for food grouping and coding in the NHNSJ may have changed with time. In addition, we cannot exclude the possibility of systematic bias in diet reporting over time, such as that from varying social desirability. Nevertheless, these factors are unlikely to explain the present findings completely, primarily because all three dietary patterns showed linear rather than abrupt changes over time.

PCA itself is subject to a number of limitations, and the results it produces might be data-specific. Our research was also hampered by analytic decisions which at several points were subjective or arbitrary. These include numbers and classifications of food groups, form of input variables, number of extracted factors, and the rotation method used, as well as the interpretation and naming of factors. Our process may have produced a degree of inconsistency, and both the results and the process used to derive dietary patterns require careful interpretation. Given the small effect size observed (i.e., regression coefficient), particularly for the “animal food and oil” and “bread and dairy” patterns, a similar analysis based on a different approach (e.g., using a diet quality score [1]) would help strengthen our understanding of trends in dietary patterns, although there is currently a lack of an appropriate diet quality score for assessing the Japanese diet [41].

In this study, misclassification of participants by weight status may have occurred because not all height and weight measurements were conducted by trained fieldworkers. Nevertheless, previous Japanese studies have consistently shown that BMI calculated from self-reported values is highly correlated with BMI calculated from measured values [50,51]. Further, similar trends in dietary patterns were generally observed across categories of weight status in this study. Finally, while food intake may differ by region or between urban and rural areas in Japan [52], we could not consider such differences in this study because of the lack of information.

## 5. Conclusions

Using national dietary survey datasets, we identified three dietary patterns in Japan, the “plant food and fish”, “bread and dairy”, and “animal food and oil” patterns. During the period of 2003–2015, the “plant food and fish” pattern score decreased while the “bread and dairy” and “animal food and oil” pattern scores increased, particularly in older participants. These findings suggest the continuous Westernization of the Japanese diet. Our results serve as both a reference and an indication for further research, as well as for the development of food-based dietary guidelines. Continuous monitoring of dietary patterns is needed to improve the diets of individuals living in Japan.

## Figures and Tables

**Table 1:** Characteristics of the 88,527 participants of the National Health and Nutrition Survey, Japan 2003–2015 ^a^.

	Year													*p* for Trend ^b^
	2003	2004	2005	2006	2007	2008	2009	2010	2011	2012	2013	2014	2015	
Sample size	7062	5675	5469	6062	5954	6198	6047	5581	5197	19,717	5393	5298	4874	
Sex, *n* (%)														0.30
Male	3129 (44.3)	2517 (44.4)	2434 (44.5)	2706 (44.6)	2682 (45.1)	2775 (44.8)	2702 (44.7)	2488 (44.6)	2330 (44.8)	8712 (44.2)	2469 (45.8)	2425 (45.8)	2188 (44.9)	
Female	3933 (55.7)	3158 (55.7)	3035 (55.5)	3356 (55.4)	3272 (55.0)	3423 (55.2)	3345 (55.3)	3093 (55.4)	2867 (55.2)	11,005 (55.8)	2924 (54.2)	2873 (54.2)	2686 (55.1)	
Age category, *n* (%)														<0.0001
20–34 years	1117 (15.8)	919 (16.2)	806 (14.7)	921 (15.2)	792 (13.3)	750 (12.1)	747 (12.4)	659 (11.8)	604 (11.6)	2111 (10.7)	579 (10.7)	516 (9.7)	462 (9.5)	
35–49 years	1630 (23.1)	1254 (22.1)	1175 (21.5)	1346 (22.2)	1449 (24.3)	1271 (20.5)	1414 (23.4)	1278 (22.9)	1181 (22.7)	4341 (22.0)	1211 (22.5)	1079 (20.4)	1098 (22.5)	
50–64 years	2117 (30.0)	1798 (31.7)	1626 (29.7)	1827 (30.1)	1768 (29.7)	1889 (30.5)	1764 (29.2)	1611 (28.9)	1492 (28.7)	5685 (28.8)	1389 (25.8)	1460 (27.6)	1306 (26.8)	
≥65 years	2198 (31.1)	1704 (30.0)	1862 (34.1)	1968 (32.5)	1945 (32.7)	2288 (36.9)	2122 (35.1)	2033 (36.4)	1920 (36.9)	7580 (38.4)	2214 (41.1)	2243 (42.3)	2008 (41.2)	
Occupation, *n* (%)														<0.0001
Professional/manager	1019 (14.4)	872 (15.4)	878 (16.1)	838 (13.8)	994 (16.7)	895 (14.4)	912 (15.1)	831 (14.9)	772 (14.9)	2745 (13.9)	828 (15.4)	726 (13.7)	772 (15.8)	
Sales/service/clerical	1701 (24.1)	1386 (24.4)	1385 (25.3)	1490 (24.6)	1451 (24.4)	1392 (22.5)	1462 (24.2)	1402 (25.1)	1262 (24.3)	4838 (24.5)	1330 (24.7)	1261 (23.8)	1195 (24.5)	
Security/transportation/labor	1585 (22.4)	1133 (20.0)	1053 (19.3)	1330 (21.9)	1121 (18.8)	1255 (20.3)	1217 (20.1)	960 (17.2)	924 (17.8)	3844 (19.5)	833 (15.5)	934 (17.6)	768 (15.8)	
Nonworker	2757 (39.0)	2284 (40.3)	2153 (39.4)	2404 (39.7)	2388 (40.1)	2656 (42.9)	2456 (40.6)	2388 (42.8)	2239 (43.1)	8290 (42.0)	2402 (44.5)	2377 (44.9)	2139 (43.9)	
Body mass index, kg/m^2^	23.0 ± 3.5	22.9 ± 3.4	23.1 ± 3.5	23.0 ± 3.4	23.0 ± 3.6	23.0 ± 3.4	23.0 ± 3.6	23.0 ± 3.5	23.0 ± 3.5	23.0 ± 3.5	22.9 ± 3.6	23.0 ± 3.5	23.0 ± 3.6	0.74
Weight status, *n* (%) ^c^														0.45
Underweight	533 (7.6)	424 (7.5)	389 (7.1)	418 (6.9)	447 (7.5)	469 (7.6)	468 (7.7)	441 (7.9)	413 (8.0)	1498 (7.6)	467 (8.7)	404 (7.6)	372 (7.6)	
Normal weight	4746 (67.2)	3848 (67.8)	3695 (67.6)	4115 (67.9)	4011 (67.4)	4220 (68.1)	4043 (66.9)	3701 (66.3)	3477 (66.9)	13,250 (67.2)	3613 (67.0)	3572 (67.4)	3320 (68.1)	
Overweight	1783 (25.3)	1403 (24.7)	1385 (25.3)	1529 (25.2)	1496 (25.1)	1509 (24.4)	1536 (25.4)	1439 (25.8)	1307 (25.2)	4969 (25.2)	1313 (24.4)	1322 (25.0)	1182 (24.3)	
Current smoking, *n* (%)														<0.0001
No	5134 (72.7)	4212 (74.2)	4176 (76.4)	4629 (76.4)	4512 (75.8)	4864 (78.5)	4650 (76.9)	4517 (80.9)	4207 (81.0)	16,135 (81.8)	4385 (81.3)	4308 (81.3)	4059 (83.3)	
Yes	1928 (27.3)	1463 (25.8)	1293 (23.6)	1433 (23.6)	1442 (24.2)	1334 (21.5)	1397 (23.1)	1064 (19.1)	990 (19.1)	3582 (18.2)	1008 (18.7)	990 (18.7)	815 (16.7)	

^a^ Values are means ± standard deviations unless otherwise indicated. ^b^ For categorical variables, a Mantel–Haenszel chi-square test was used; for continuous variables, a linear trend test was used with the survey year as a continuous variable in linear regression. ^c^ Defined based on body mass index (kg/m^2^): <18.5 for underweight, ≥18.5 to <25 for normal weight, and ≥25 for overweight (including obese).

**Table 2:** Factor loadings for dietary patterns identified among the 88,527 participants of the National Health and Nutrition Survey, Japan 2003–2015 ^a^.

	Factor 1 “Plant Food and Fish” Pattern	Factor 2 “Bread and Dairy” Pattern	Factor 3 “Animal Food and Oil” Pattern
Rice	**0.34**	**−0.55**	0.16
Bread	−0.19	**0.64**	0.16
Noodles	−0.21	0.04	0.02
Other grains	0.00	0.06	0.25
Potatoes	**0.36**	0.00	0.16
Sugar	**0.33**	**0.34**	0.09
Pulses	**0.41**	−0.03	−0.07
Nuts	0.18	0.17	−0.03
Green and yellow vegetables	**0.50**	0.19	0.07
Other vegetables	**0.48**	0.01	**0.33**
Vegetable and fruit juice	−0.03	0.15	0.04
Pickled vegetables	**0.30**	−0.14	−0.09
Fruit	**0.43**	**0.40**	−0.22
Mushrooms	**0.30**	0.04	0.08
Seaweeds	**0.30**	−0.01	−0.04
Fish	**0.31**	−0.04	−0.15
Shellfish	0.08	−0.06	0.17
Sea products	0.27	−0.12	−0.04
Red meat	0.02	−0.10	**0.48**
Processed meat	−0.09	0.12	**0.37**
Chicken	−0.01	−0.05	0.24
Eggs	0.12	−0.03	**0.39**
Dairy products	0.15	**0.54**	−0.08
Animal fat	−0.09	0.29	0.25
Vegetable oil	0.00	0.11	**0.64**
Confectioneries	−0.01	0.23	−0.08
Alcoholic beverages	−0.03	−0.24	0.28
Tea	**0.34**	0.05	−0.22
Coffee	−0.12	0.23	0.28
Soft drinks	−0.12	0.00	0.21
Salt-based seasonings	**0.60**	−0.22	0.16
Variability explained (%)	7.43	5.64	5.57

^a^ Dietary patterns were identified using principal component analysis based on intakes of the 31 food groups (g/day). Absolute factor loading values ≥0.30 are presented in bold. Total variability explained was 18.63%.

## Supplementary Materials

The following are available online at http://www.mdpi.com/2072-6643/10/8/994/s1, Table S1: Food groups used in the present study, Table S2: Characteristics of the participants included in the analysis and those excluded from the analysis, Table S3: Food group intake (g/day) among the participants included in the analysis and those excluded from the analysis, Table S4: Thirteen-year trends (2003–2015) in food group intake (g/day): National Health and Nutrition Survey, Japan, Table S5: Thirteen-year trends (2003–2015) in the “plant food and fish” dietary pattern score (factor 1): National Health and Nutrition Survey, Japan, Table S6: Thirteen-year trends (2003–2015) in the “bread and dairy” dietary pattern score (factor 2): National Health and Nutrition Survey, Japan, Table S7: Thirteen-year trends (2003–2015) in the “animal food and oil” dietary pattern score (factor 3): National Health and Nutrition Survey, Japan, Table S8: Factor loadings for dietary patterns identified among the 68,810 participants of the National Health and Nutrition Survey, Japan 2003–2011 and 2013–2015 (excluding 2012 data), Table S9: Secular trends in the “plant food and fish” dietary pattern score (factor 1): National Health and Nutrition Survey, Japan 2003–2011 and 2013–2015 (excluding 2012 data), Table S10: Secular trends in the “bread and dairy” dietary pattern score (factor 2): National Health and Nutrition Survey, Japan 2003–2011 and 2013–2015 (excluding 2012 data), Table S11: Secular trends in the “animal food and oil” dietary pattern score (factor 3): National Health and Nutrition Survey, Japan 2003–2011 and 2013–2015 (excluding 2012 data).
