# Supplementary material for: Thirteen-Year Trends in Dietary Patterns among Japanese Adults in the National Health and Nutrition Survey 2003–2015: Continuous Westernization of the Japanese Diet
Source: Nutrients. 2018 Jul 30;10(8):994. doi: 10.3390/nu10080994 (PMC6115850; doi:10.3390/nu10080994)
Supplement: Supplementary file 1 [file nutrients-10-00994-s001.pdf]

Table S1. Food groups used in this study [a]

Food groups	Food items used in the National Health and Nutrition Survey, Japan [food number]
Rice	Rice [1], rice products [2]
Bread	Bread [4]
Noodles	Japanese wheat noodles and Chinese noodles [6], instant noodles [7], pasta [8], buckwheat noodles [10]
Other grains	Wheat flour [3], wheat products [9], corn products [11], other grains [12]
Potatoes	Sweet potatoes [13], potatoes [14], other potatoes [15], processed starch [16]
Sugar	Sugars and sweeteners [17], jam [44]
Pulses	Soybeans [18], tofu [19], fried tofu [20], natto (fermented soybeans) [21], other soybean products [22], other beans [23]
Nuts	Nuts [24]
Green and yellow vegetables	Tomatoes [25], carrots [26], spinach [27], green peppers [28], other green and yellow vegetables [29]
Other vegetables	Cabbage [30], cucumbers [31], radishes [32], onions [33], Chinese cabbage [34], other vegetables [35]
Vegetable and fruit juice	Vegetable juice [36], fruit juice [45]
Pickled vegetables	Pickled leafy vegetables [37], other picked vegetables [38]
Fruit	Strawberries [39], citrus fruits [40], bananas [41], apples [42], other fruits [43]
Mushrooms	Mushrooms [46]
Seaweeds	Seaweeds [47]
Fish	Mackerel and sardine [48], salmon and trout [49], sea bream and flat fish [50], tuna and swordfish [51], other fish [52]
Shellfish	Shellfish [53], squid and octopus [54], prawn and crab [55]
Sea products	Processed fish and shellfish [56], canned fish and shellfish [57], boiled fish and shellfish in soy sauce [58], fish paste [59], fish products [60]
Red meat	Beef [61], port [62], other red meat [64], offal [67], whale [68], boiled meat in soy sauce [69]
Processed meat	Ham and sausage [63]
Chicken	Chicken [65], other poultry [66]
Eggs	Eggs [70]
Dairy products	Milk [71], cheese [72], yogurt [73], other dairy products [74], other milks [75]
Animal fat	Butter [76], animal fats [79]
Vegetable oil	Margarine [77], vegetable oils [78], other oils [80], mayonnaise [95]
Confectioneries	Bread with a sweet filling [5], Japanese confectioneries [81], cakes and pastries [82], biscuits [83], candies [84], other confectioneries [85]
Alcoholic beverages	Sake [86], beer [87], wine and other alcoholic beverages [88]
Tea	Green tea, oolong tea, and black tea [89]
Coffee	Coffee and cocoa [90]
Soft drinks	Sugar-sweetened beverages and other beverages [91]
Salt-based seasonings	Sauce [92], soy sauce [93], salt [94], miso [96]

[a] In total, 96 of 98 food items used in the National Health and Nutrition Survey, Japan were categorized into 31 food groups as shown above, while the 2 remaining items (other seasonings [97] and spices [98]) were not used in this analysis.

Table S2. Characteristics of the participants included in the analysis and those excluded from the analysis [a]

	Participants included in the analysis (n = 88,527)	Participants excluded from the analysis (n = 23,255)[b]	P[c]
Year, n (%)			<0.0001
2003	7062 (8.0)	1785 (7.7)	
2004	5675 (6.4)	1291 (5.6)	
2005	5469 (6.2)	1720 (7.4)	
2006	6062 (6.9)	1443 (6.2)	
2007	5954 (6.7)	1149 (4.9)	
2008	6198 (7.0)	1375 (5.9)	
2009	6047 (6.8)	1238 (5.3)	
2010	5581 (6.3)	1555 (6.7)	
2011	5197 (5.9)	1468 (6.3)	
2012	19,717 (22.3)	6644 (28.6)	
2013	5393 (6.1)	1017 (4.4)	
2014	5298 (6.0)	1355 (5.8)	
2015	4874 (5.5)	1215 (5.2)	
Sex, n (%)			<0.0001
Male	39,557 (44.7)	12,515 (53.8)	
Female	48,970 (55.3)	10,740 (46.2)	
Age, years	56.4 ± 16.8	50.9 ± 19.9	<0.0001
Age category, n (%)			<0.0001
20-34 years	10,983 (12.4)	5827 (25.1)	
35-49 years	19,727 (22.3)	5686 (24.5)	
50-64 years	25,732 (29.1)	5409 (23.3)	
≥65 years	32,085 (36.2)	6333 (27.2)	
Occupation, n (%)			<0.0001
Professional/manager	13,082 (14.8)	3591 (16.8)	
Sales/service/clerical	21,555 (24.4)	5876 (27.5)	
Security/transportation/labor	16,957 (19.2)	4698 (22.0)	
Nonworker	36,933 (41.7)	7228 (33.8)	
Body mass index, kg/m^2	23.0 ± 3.5	21.9 ± 3.5	<0.0001
Weight status, n (%)[d]			<0.0001
Underweight	6743 (7.6)	275 (13.2)	
Normal weight	59,611 (67.3)	1457 (70.1)	
Overweight	22,173 (25.1)	346 (16.7)	
Current smoking, n (%)			<0.0001
No	69,788 (78.8)	15,561 (73.0)	
Yes	18,739 (21.2)	5751 (27.0)	

[a] Values are means ± standard deviations unless otherwise indicated.

[b] n = 21,393 for occupation, n = 2078 for body mass index (BMI) and weight status, and n = 21,312 for current smoking (because of missing information).

[c] P values for difference between participants included in the analysis and those excluded from the analysis were based on the chi-square test for categorical variables and independent t-test for continuous variables.

[d] Defined based on BMI (kg/m^2): <18.5 for underweight, ≥18.5 to <25 for normal weight, and ≥25 for overweight (including obese).

Table S3. Food group intake (g/day) among the participants included in the analysis and those excluded from the analysis [a]

	Participants included in the analysis (n = 88,527)	Participants excluded from the analysis (n = 23,255)	P^b
Rice	341.8 ± 186.0	351.6 ± 191.4	<0.0001
Bread	31.6 ± 43.9	28.9 ± 44.6	<0.0001
Noodles	62.2 ± 101.6	62.9 ± 103.5	0.36
Other grains	10.6 ± 32.3	10.6 ± 32.1	0.87
Potatoes	57.2 ± 70.6	53.8 ± 69.0	<0.0001
Sugar	8.5 ± 10.6	7.1 ± 9.6	<0.0001
Pulses	63.4 ± 77.4	55.1 ± 71.2	<0.0001
Nuts	2.3 ± 8.6	1.5 ± 6.4	<0.0001
Green and yellow vegetables	97.8 ± 88.6	84.8 ± 79.1	<0.0001
Other vegetables	172.8 ± 123.1	160.3 ± 117.6	<0.0001
Vegetable and fruit juice	17.8 ± 66.3	18.4 ± 71.0	0.23
Pickled vegetables	15.2 ± 29.3	12.8 ± 25.4	<0.0001
Fruit	110.9 ± 135.2	81.1 ± 119.3	<0.0001
Mushrooms	17.3 ± 28.8	15.3 ± 26.9	<0.0001
Seaweeds	12.1 ± 23.9	10.4 ± 22.7	<0.0001
Fish	38.3 ± 55.5	35.8 ± 53.9	<0.0001
Shellfish	14.1 ± 33.1	14.3 ± 32.5	0.36
Sea products	31.4 ± 45.4	28.6 ± 43.1	<0.0001
Red meat	46.3 ± 56.3	51.1 ± 60.8	<0.0001
Processed meat	11.5 ± 21.4	12.2 ± 21.8	<0.0001
Chicken	20.9 ± 42.4	23.5 ± 45.4	<0.0001
Eggs	34.6 ± 34.2	35.4 ± 36.4	0.0007
Dairy products	100.7 ± 130.6	82.6 ± 126.7	<0.0001
Animal fat	1.0 ± 3.0	1.1 ± 3.2	<0.0001
Vegetable oil	12.0 ± 11.4	12.5 ± 11.8	<0.0001
Confectioneries	29.4 ± 50.9	25.4 ± 49.6	<0.0001
Alcoholic beverages	122.9 ± 290.3	112.6 ± 278.6	<0.0001
Tea	341.3 ± 372.2	262.8 ± 328.4	<0.0001
Coffee	157.5 ± 208.0	125.7 ± 190.3	<0.0001
Soft drinks	93.6 ± 224.6	87.5 ± 227.6	0.0003
Salt-based seasonings	31.9 ± 20.9	30.4 ± 20.5	<0.0001

[a] Values are means ± standard deviations.

[b] P values for difference between participants included in the analysis and those excluded from the analysis were based on an independent t-test.

Table S4. Thirteen-year trends (2003-2015) in food group intake (g/day): National Health and Nutrition Survey, Japan [a]

	Year													*P* for	Per-year change
	2003	2004	2005	2006	2007	2008	2009	2010	2011	2012	2013	2014	2015	trend[b]	β ± SE[c]
Sample size	7062	5675	5469	6062	5954	6198	6047	5581	5197	19,717	5393	5298	4874		
Rice	363.9 ± 2.0	354.7 ± 2.3	351.5 ± 2.3	351.5 ± 2.2	343.9 ± 2.2	346.9 ± 2.2	341.9 ± 2.2	335.9 ± 2.3	327.8 ± 2.4	343.4 ± 1.2	324.2 ± 2.3	324.9 ± 2.3	316.1 ± 2.4	<0.0001	-2.94 ± 0.16
Bread	28.2 ± 0.5	29.7 ± 0.6	30.4 ± 0.6	29.7 ± 0.6	30.7 ± 0.6	31.4 ± 0.6	33.2± 0.6	32.3 ± 0.6	32.3 ± 0.6	30.9 ± 0.3	35.3 ± 0.6	34.1 ± 0.6	36.2 ± 0.6	<0.0001	0.45 ± 0.04
Noodles	61.4 ± 1.2	60.1 ± 1.3	62.4 ± 1.4	60.0 ± 1.3	60.5 ± 1.3	62.0 ± 1.3	63.4 ± 1.3	61.5 ± 1.4	65.3 ± 1.4	61.6 ± 0.7	65.6 ± 1.4	64.6 ± 1.4	63.2 ± 1.4	0.005	0.27 ± 0.09
Other grains	11.0 ± 0.4	9.4 ± 0.4	10.8 ± 0.4	11.4 ± 0.4	10.4 ± 0.4	11.0 ± 0.4	10.0 ± 0.4	10.5 ± 0.4	9.5 ± 0.4	10.5 ± 0.2	10.3 ± 0.4	11.6 ± 0.4	11.7 ± 0.5	0.37	0.03 ± 0.03
Potatoes	61.3 ± 0.8	60.7 ± 0.9	59.9 ± 0.9	64.1 ± 0.9	58.3 ± 0.9	58.2 ± 0.9	55.4 ± 0.9	54.3 ± 0.9	55.3 ± 1.0	56.9 ± 0.5	52.6 ± 1.0	53.3 ± 1.0	51.3 ± 1.0	<0.0001	-0.78 ± 0.07
Sugar	9.1 ± 0.1	9.0 ± 0.1	9.1 ± 0.1	9.0 ± 0.1	8.5 ± 0.1	8.4 ± 0.1	8.4 ± 0.1	8.5 ± 0.1	8.3 ± 0.1	8.2 ± 0.1	8.2 ± 0.1	8.1 ± 0.1	8.4 ± 0.2	<0.0001	-0.09 ± 0.01
Pulses	65.6 ± 0.9	67.6 ± 1.0	64.4 ± 1.0	62.5 ± 1.0	62.4 ± 1.0	60.7 ± 1.0	61.6 ± 1.0	61.8 ± 1.0	56.5 ± 1.0	64.7 ± 0.5	65.3 ± 1.0	62.3 ± 1.1	65.3 ± 1.1	0.15	-0.10 ± 0.07
Nuts	2.5 ± 0.1	2.4 ± 0.1	2.2 ± 0.1	2.4 ± 0.1	2.3 ± 0.1	2.0 ± 0.1	2.1 ± 0.1	2.4 ± 0.1	2.3 ± 0.1	2.5 ± 0.1	2.1 ± 0.1	2.2 ± 0.1	2.6 ± 0.1	0.79	-0.002 ± 0.008
Green and yellow vegetables	106.6 ± 1.0	92.8 ± 1.2	103.0 ± 1.2	106.4 ± 1.1	101.4 ± 1.1	99.9 ± 1.1	102.9 ± 1.1	95.9 ± 1.2	93.8 ± 1.2	93.5 ± 0.6	87.7 ± 1.2	93.3 ± 1.2	100.2 ± 1.2	<0.0001	-0.97 ± 0.08
Other vegetables	172.3 ± 1.5	154.4 ± 1.6	172.7 ± 1.6	177.4 ± 1.6	170.2 ± 1.6	174.9 ± 1.5	174.9 ± 1.6	168.7 ± 1.6	165.8 ± 1.7	177.6 ± 0.9	173.9 ± 1.7	178.0 ± 1.7	173.7 ± 1.7	<0.0001	0.74 ± 0.11
Vegetable and fruit juice	13.4 ± 0.8	16.1 ± 0.9	16.1 ± 0.9	17.7 ± 0.9	17.3 ± 0.8	19.5 ± 0.9	16.3 ± 0.8	16.6 ± 0.9	17.5 ± 0.9	18.2 ± 0.9	20.2 ± 0.9	19.0 ± 0.9	21.0 ± 0.9	<0.0001	0.40 ± 0.06
Pickled vegetables	21.6 ± 0.3	19.9 ± 0.4	19.1 ± 0.4	17.7 ± 0.4	16.4 ± 0.4	17.1 ± 0.4	15.7 ± 0.4	13.6 ± 0.4	14.1 ± 0.4	13.2 ± 0.2	11.0 ± 0.4	10.6 ± 0.4	10.2 ± 0.4	<0.0001	-0.92 ± 0.03
Fruit	118.1 ± 1.5	120.8 ± 1.7	127.5 ± 1.7	110.9 ± 1.6	114.1 ± 1.6	117.9 ± 1.6	112.6 ± 1.6	100.6 ± 1.7	105.9 ± 1.8	106.4 ± 0.9	108.5 ± 1.7	100.8 ± 1.7	104.8 ± 1.8	<0.0001	-1.64 ± 0.12
Mushrooms	17.0 ± 0.3	16.9 ± 0.4	17.8 ± 0.4	16.4 ± 0.4	17.4 ± 0.4	16.6 ± 0.4	17.0 ± 0.4	18.1 ± 0.4	16.0 ± 0.4	18.1 ± 0.2	17.7 ± 0.4	16.5 ± 0.4	17.0 ± 0.4	0.08	0.05 ± 0.03
Seaweeds	14.6 ± 0.3	14.3 ± 0.3	16.2 ± 0.3	14.0 ± 0.3	12.7 ± 0.3	10.8 ± 0.3	11.1 ± 0.3	12.1 ± 0.3	11.2 ± 0.3	10.8 ± 0.2	10.8 ± 0.3	10.1 ± 0.3	11.0 ± 0.3	<0.0001	-0.42 ± 0.02
Fish	44.4 ± 0.7	43.6 ± 0.7	43.3 ± 0.7	42.2 ± 0.7	40.6 ± 0.7	36.7 ± 0.7	37.0 ± 0.7	35.4 ± 0.7	36.6 ± 0.8	36.5 ± 0.4	35.9 ± 0.7	33.8 ± 0.8	34.3 ± 0.8	<0.0001	-0.89 ± 0.05
Shellfish	17.1 ± 0.4	17.1 ± 0.4	16.3 ± 0.4	15.5 ± 0.4	15.2 ± 0.4	15.0 ± 0.4	14.1 ± 0.4	12.8 ± 0.4	13.6 ± 0.5	12.5 ± 0.2	12.4 ± 0.4	12.3 ± 0.5	11.2 ± 0.5	<0.0001	-0.49 ± 0.03
Sea products	34.8 ± 0.5	32.8 ± 0.6	33.1 ± 0.6	30.1 ± 0.6	32.0 ± 0.6	32.8 ± 0.6	30.5 ± 0.6	31.4 ± 0.6	31.1 ± 0.6	30.7 ± 0.3	31.6 ± 0.6	28.0 ± 0.6	29.1 ± 0.6	<0.0001	-0.36 ± 0.04

Red meat	41.5 ± 0.7	44.7 ± 0.7	46.1 ± 0.7	43.5 ± 0.7	47.0 ± 0.7	46.0 ± 0.7	46.0 ± 0.7	46.0 ± 0.7	44.2 ± 0.8	47.1 ± 0.4	50.4 ± 0.7	48.6 ± 0.8	50.8 ± 0.8	<0.0001	0.52 ± 0.05
Processed meat	10.4 ± 0.3	10.4 ± 0.3	10.9 ± 0.3	11.0 ± 0.3	10.8 ± 0.3	10.2 ± 0.3	11.9 ± 0.3	11.4 ± 0.3	12.1 ± 0.3	12.1 ± 0.2	12.4 ± 0.3	13.3 ± 0.3	12.3 ± 0.3	<0.0001	0.21 ± 0.02
Chicken	18.6 ± 0.5	17.6 ± 0.6	17.8 ± 0.6	18.6 ± 0.5	18.9 ± 0.5	18.0 ± 0.5	20.2 ± 0.5	21.1 ± 0.6	21.6 ± 0.6	22.6 ± 0.3	24.2 ± 0.6	25.8 ± 0.6	24.4 ± 0.6	<0.0001	0.65 ± 0.04
Eggs	35.5 ± 0.4	33.4 ± 0.5	34.2 ± 0.5	35.7 ± 0.4	35.1 ± 0.4	32.4 ± 0.4	34.3 ± 0.4	35.0 ± 0.5	34.2 ± 0.5	34.7 ± 0.2	34.2 ± 0.5	34.9 ± 0.5	35.3 ± 0.5	0.67	0.01 ± 0.03
Dairy products	103.9 ± 1.5	104.9 ± 1.7	102.4 ± 1.7	99.8 ± 1.7	100.9 ± 1.7	91.9 ± 1.6	93.8 ± 1.7	95.4 ± 1.7	99.2 ± 1.8	100.2 ± 0.9	103.1 ± 1.8	104.3 ± 1.8	112.4 ± 1.8	0.17	0.17 ± 0.12
Animal fat	1.0 ± 0.04	1.0 ± 0.04	1.0 ± 0.04	1.0 ± 0.04	1.1 ± 0.04	1.0 ± 0.04	1.0 ± 0.04	1.0 ± 0.04	1.0 ± 0.04	0.9 ± 0.02	1.1 ± 0.04	1.1 ± 0.04	1.2 ± 0.04	0.87	0.0005 ± 0.003
Vegetable oil	12.0 ± 0.1	12.0 ± 0.1	11.8 ± 0.1	11.8 ± 0.1	12.1 ± 0.1	11.1 ± 0.1	12.0 ± 0.1	12.1 ± 0.1	11.8 ± 0.2	11.8 ± 0.1	12.0 ± 0.2	12.9 ± 0.2	12.9 ± 0.2	0.0001	0.04 ± 0.01
Confectioneries	30.2 ± 0.6	28.7 ± 0.7	29.0 ± 0.7	29.6 ± 0.7	29.2 ± 0.7	31.9 ± 0.6	27.7 ± 0.7	28.6 ± 0.7	28.7 ± 0.7	28.8 ± 0.4	30.9 ± 0.7	30.6 ± 0.7	30.0 ± 0.7	0.84	0.01 ± 0.05
Alcoholic beverages	117.2 ± 3.3	122.6 ± 3.6	113.2 ± 3.7	121.3 ± 3.5	123.7 ± 3.6	118.3 ± 3.5	120.5 ± 3.5	118.2 ± 3.7	130.6 ± 3.8	123.8 ± 2.0	126.2 ± 3.7	131.6 ± 3.8	133.3 ± 3.9	<0.0001	1.05 ± 0.26
Tea	370.6 ± 4.3	378.2 ± 4.8	378.0 ± 4.9	378.8 ± 4.7	364.8 ± 4.7	345.8 ± 4.6	365.6 ± 4.7	331.7 ± 4.9	353.0 ± 5.1	309.6 ± 2.6	300.4 ± 5.0	296.3 ± 5.0	323.6 ± 5.2	<0.0001	-7.30 ± 0.34
Coffee	142.3 ± 2.4	149.5 ± 2.7	161.3 ± 2.7	145.3 ± 2.6	154.4 ± 2.6	146.3 ± 2.6	155.9 ± 2.6	158.3 ± 2.7	155.4 ± 2.8	160.6 ± 1.4	170.6 ± 2.7	166.5 ± 2.8	183.4 ± 2.9	<0.0001	2.21 ± 0.19
Soft drinks	55.4 ± 2.6	68.3 ± 2.9	64.6 ± 3.0	79.4 ± 2.8	85.4 ± 2.8	67.8 ± 2.8	87.2 ± 2.8	89.4 ± 2.9	102.3 ± 3.0	98.5 ± 1.6	92.3 ± 3.0	86.1 ± 3.0	264.0 ± 3.1	<0.0001	7.42 ± 0.21[d]
Salt-based seasonings	37.6 ± 0.2	35.4 ± 0.3	36.7 ± 0.3	36.2 ± 0.3	33.3 ± 0.3	32.6 ± 0.3	31.6 ± 0.3	31.3 ± 0.3	29.6 ± 0.3	29.7 ± 0.1	28.2 ± 0.3	27.6 ± 0.3	26.7 ± 0.3	<0.0001	-0.91 ± 0.02

[a] Values are means ± standard errors unless otherwise indicated. Adjustment was made for sex, age category, occupation, weight status, and current smoking.

[b] A linear trend test was used with the survey year as a continuous variable in linear regression.

[c] *P* values for regression coefficients are theoretically identical to *P* values for trend.

[d] The analysis was repeated after excluding the 2015 data because of the extremely high mean value, but a significant result remained: 3.59 ± 0.21 (P <0.0001).

Table S5. Thirteen-year trends (2003-2015) in the “plant food and fish” dietary pattern score (factor 1): National Health and Nutrition Survey, Japan [a]

		Year													*P* for	Per-year change
	n	2003	2004	2005	2006	2007	2008	2009	2010	2011	2012	2013	2014	2015	trend [b]	β ± SE [c]
All	88,527	0.25 ± 0.01	0.13 ± 0.01	0.20 ± 0.01	0.17 ± 0.01	0.06 ± 0.01	0.03 ± 0.01	0.00 ± 0.01	-0.07 ± 0.01	-0.12 ± 0.01	-0.07 ± 0.01	-0.16 ± 0.01	-0.18 ± 0.01	-0.20 ± 0.01	<0.0001	-0.036 ± 0.001
Sex (*P* for interaction = 0.85)[d]																
Male	39,557	0.35 ± 0.02	0.21 ± 0.02	0.28 ± 0.02	0.26 ± 0.02	0.14 ± 0.02	0.13 ± 0.02	0.08 ± 0.02	0.02 ± 0.02	-0.04 ± 0.02	0.02 ± 0.01	-0.06 ± 0.02	-0.09 ± 0.02	-0.14 ± 0.02	<0.0001	-0.037 ± 0.001
Female	48,970	0.16 ± 0.01	0.06 ± 0.02	0.14 ± 0.02	0.09 ± 0.02	-0.01 ± 0.02	-0.05 ± 0.02	-0.07 ± 0.02	-0.14 ± 0.02	-0.17 ± 0.02	-0.14 ± 0.01	-0.24 ± 0.02	-0.26 ± 0.02	-0.24 ± 0.02	<0.0001	-0.036 ± 0.001
Age category (*P* for interaction = 0.06)[d]																
20-34 years	10,983	-0.31 ± 0.02	-0.34 ± 0.03	-0.33 ± 0.03	-0.40 ± 0.03	-0.44 ± 0.03	-0.49 ± 0.03	-0.48 ± 0.03	-0.52 ± 0.03	-0.60 ± 0.03	-0.53 ± 0.02	-0.58 ± 0.03	-0.63 ± 0.04	-0.59 ± 0.04	<0.0001	-0.026 ± 0.002
35-49 years	19,727	-0.15 ± 0.02	-0.30 ± 0.02	-0.22 ± 0.02	-0.23 ± 0.02	-0.40 ± 0.02	-0.41 ± 0.02	-0.40 ± 0.02	-0.50 ± 0.02	-0.51 ± 0.02	-0.47 ± 0.01	-0.57 ± 0.02	-0.55 ± 0.03	-0.56 ± 0.02	<0.0001	-0.033 ± 0.002
50-64 years	25,732	0.41 ± 0.02	0.30 ± 0.02	0.36 ± 0.02	0.28 ± 0.02	0.23 ± 0.02	0.17 ± 0.02	0.09 ± 0.02	0.04 ± 0.02	-0.03 ± 0.02	-0.01 ± 0.01	-0.12 ± 0.03	-0.12 ± 0.03	-0.21 ± 0.03	<0.0001	-0.049 ± 0.002
≥65 years	32,085	0.55 ± 0.02	0.40 ± 0.02	0.52 ± 0.02	0.52 ± 0.02	0.39 ± 0.02	0.35 ± 0.02	0.33 ± 0.02	0.27 ± 0.02	0.23 ± 0.02	0.28 ± 0.01	0.20 ± 0.02	0.15 ± 0.02	0.17 ± 0.02	<0.0001	-0.032 ± 0.002
Occupation (*P* for interaction = 0.53)[d]																
Professional/manager	13,082	0.13 ± 0.03	0.03 ± 0.03	0.09 ± 0.03	0.05 ± 0.03	-0.07 ± 0.03	-0.12 ± 0.03	-0.07 ± 0.03	-0.12 ± 0.03	-0.21 ± 0.03	-0.15 ± 0.02	-0.28 ± 0.03	-0.24 ± 0.03	-0.28 ± 0.03	<0.0001	-0.033 ± 0.002
Sales/service/clerical	21,555	0.01 ± 0.02	-0.07 ± 0.02	-0.05 ± 0.02	-0.06 ± 0.02	-0.16 ± 0.02	-0.18 ± 0.02	-0.24 ± 0.02	-0.29 ± 0.02	-0.36 ± 0.02	-0.30 ± 0.01	-0.40 ± 0.02	-0.39 ± 0.02	-0.42 ± 0.03	<0.0001	-0.036 ± 0.002

Security/transportation/ labor	16,957	0.41 ± 0.02	0.27 ± 0.03	0.32 ± 0.03	0.30 ± 0.03	0.18 ± 0.03	0.20 ± 0.03	0.13 ± 0.03	0.02 ± 0.03	0.00 ± 0.03	0.03 ± 0.02	-0.09 ± 0.03	-0.11 ± 0.03	-0.20 ± 0.04	<0.0001	-0.044 ± 0.002
Non-worker	36,933	0.34 ± 0.02	0.22 ± 0.02	0.33 ± 0.02	0.28 ± 0.02	0.19 ± 0.02	0.12 ± 0.02	0.10 ± 0.02	0.04 ± 0.02	0.01 ± 0.02	0.05 ± 0.01	-0.01 ± 0.02	-0.08 ± 0.02	-0.05 ± 0.02	<0.0001	-0.034 ± 0.001
Weight status (*P* for interaction = 0.0001)[d,e]																
Underweight	6743	-0.05 ± 0.04	-0.20 ± 0.04	-0.06 ± 0.04	-0.03 ± 0.04	-0.17 ± 0.04	-0.23 ± 0.04	-0.25 ± 0.04	-0.29 ± 0.04	-0.32 ± 0.04	-0.26 ± 0.02	-0.38 ± 0.04	-0.34 ± 0.04	-0.39 ± 0.04	<0.0001	-0.026 ± 0.003
Normal weight	59,611	0.24 ± 0.01	0.13 ± 0.01	0.21 ± 0.01	0.17 ± 0.01	0.04 ± 0.01	0.03 ± 0.01	0.00 ± 0.01	-0.06 ± 0.01	-0.13 ± 0.02	-0.08 ± 0.01	-0.16 ± 0.02	-0.18 ± 0.02	-0.20 ± 0.02	<0.0001	-0.037 ± 0.001
Overweight	22,173	0.35 ± 0.02	0.22 ± 0.03	0.26 ± 0.03	0.23 ± 0.02	0.17 ± 0.03	0.12 ± 0.03	0.08 ± 0.02	-0.03 ± 0.03	-0.03 ± 0.03	0.02 ± 0.01	-0.08 ± 0.03	-0.15 ± 0.03	-0.14 ± 0.03	<0.0001	-0.039 ± 0.002
Current smoking (*P* for interaction = 0.46)[d]																
No	69,788	0.33 ± 0.01	0.19 ± 0.01	0.28 ± 0.01	0.23 ± 0.01	0.12 ± 0.01	0.10 ± 0.01	0.07 ± 0.01	0.00 ± 0.01	-0.04 ± 0.01	-0.01 ± 0.01	-0.08 ± 0.01	-0.12 ± 0.01	-0.13 ± 0.01	<0.0001	-0.037 ± 0.001
Yes	18,739	-0.06 ± 0.02	-0.10 ± 0.02	-0.09 ± 0.03	-0.07 ± 0.02	-0.17 ± 0.02	-0.22 ± 0.02	-0.26 ± 0.02	-0.31 ± 0.03	-0.38 ± 0.03	-0.30 ± 0.02	-0.47 ± 0.03	-0.42 ± 0.03	-0.47 ± 0.03	<0.0001	-0.035 ± 0.002

[a] Values are mean ± standard error scores unless otherwise indicated. The dietary pattern score represents standardized variables with mean 0 and standard deviation 1. Negative scores indicate low adherence to the dietary pattern, whereas positive scores indicate high adherence. Adjustment was made for sex, age category, occupation, weight status, and current smoking. The number of participants in each category is shown in Table 1.

[b] A linear trend test was used with the survey year as a continuous variable in linear regression.

[c] *P* values for regression coefficients are theoretically identical to *P* values for trend.

[d] To calculate *P* values for interaction, the product term of the time and variable of stratification was added into the linear regression model.

[e] Defined based on body mass index (kg/m^2): <18.5 for underweight, ≥18.5 to <25 for normal weight, and ≥25 for overweight (including obese).

Table S6. Thirteen-year trends (2003-2015) in the “bread and dairy” dietary pattern score (factor 2): National Health and Nutrition Survey, Japan [a]

		Year													*P* for	Per-year change
	n	2003	2004	2005	2006	2007	2008	2009	2010	2011	2012	2013	2014	2015	trend[b]	β ± SE[c]
All	88,527	-0.08 ± 0.01	-0.05 ± 0.01	-0.01 ± 0.01	-0.04 ± 0.01	0.00 ± 0.01	-0.05 ± 0.01	-0.01 ± 0.01	0.00 ± 0.01	0.02 ± 0.01	-0.02 ± 0.01	0.08 ± 0.01	0.08 ± 0.01	0.17 ± 0.01	<0.0001	0.012 ± 0.001
Sex (*P* for interaction = 0.009)[d]																
Male	39,557	-0.34 ± 0.02	-0.31 ± 0.02	-0.28 ± 0.02	-0.29 ± 0.02	-0.26 ± 0.02	-0.30 ± 0.02	-0.27 ± 0.02	-0.27 ± 0.02	-0.26 ± 0.02	-0.29 ± 0.01	-0.19 ± 0.02	-0.18 ± 0.02	-0.09 ± 0.02	<0.0001	0.011 ± 0.001
Female	48,970	0.14 ± 0.01	0.17 ± 0.02	0.22 ± 0.02	0.16 ± 0.02	0.21 ± 0.02	0.16 ± 0.02	0.20 ± 0.02	0.21 ± 0.02	0.24 ± 0.02	0.20 ± 0.01	0.30 ± 0.02	0.28 ± 0.02	0.37 ± 0.02	<0.0001	0.012 ± 0.001
Age category (*P* for interaction <0.0001)[d]																
20-34 years	10,983	-0.15 ± 0.03	-0.16 ± 0.03	-0.12 ± 0.03	-0.07 ± 0.03	-0.13 ± 0.03	-0.19 ± 0.03	-0.17 ± 0.03	-0.15 ± 0.04	-0.11 ± 0.04	-0.21 ± 0.02	-0.18 ± 0.04	-0.09 ± 0.04	-0.13 ± 0.04	0.19	-0.003 ± 0.002
35-49 years	19,727	-0.13 ± 0.02	-0.10 ± 0.03	-0.09 ± 0.03	-0.10 ± 0.03	-0.09 ± 0.02	-0.13 ± 0.03	-0.07 ± 0.02	-0.06 ± 0.03	-0.09 ± 0.03	-0.13 ± 0.01	-0.09 ± 0.03	-0.07 ± 0.03	-0.02 ± 0.03	0.11	0.003 ± 0.002
50-64 years	25,732	-0.02 ± 0.02	-0.03 ± 0.02	0.07 ± 0.02	-0.02 ± 0.02	0.09 ± 0.02	0.00 ± 0.02	0.04 ± 0.02	0.00 ± 0.02	0.05 ± 0.03	0.02 ± 0.01	0.10 ± 0.03	0.10 ± 0.03	0.22 ± 0.03	<0.0001	0.009 ± 0.002
≥65 years	32,085	-0.08 ± 0.02	0.01 ± 0.02	0.02 ± 0.02	-0.03 ± 0.02	0.02 ± 0.02	0.02 ± 0.02	0.02 ± 0.02	0.08 ± 0.02	0.11 ± 0.02	0.08 ± 0.01	0.24 ± 0.02	0.19 ± 0.02	0.32 ± 0.02	<0.0001	0.024 ± 0.002
Occupation (*P* for interaction <0.0001)[d]																
Professional/manager	13,082	-0.03 ± 0.03	-0.01 ± 0.03	0.00 ± 0.03	-0.07 ± 0.03	0.01 ± 0.03	-0.07 ± 0.03	-0.02 ± 0.03	-0.01 ± 0.03	-0.03 ± 0.03	-0.06 ± 0.02	-0.01 ± 0.03	0.02 ± 0.04	0.04 ± 0.03	0.63	0.001 ± 0.002
Sales/service/clerical	21,555	-0.02 ± 0.02	-0.03 ± 0.03	0.04 ± 0.03	0.02 ± 0.02	0.02 ± 0.02	-0.04 ± 0.03	0.00 ± 0.02	-0.01 ± 0.03	-0.02 ± 0.03	-0.02 ± 0.01	0.03 ± 0.03	0.03 ± 0.03	0.15 ± 0.03	0.01	0.004 ± 0.002

Security/transportation/ Labor	16,957	-0.50 ± 0.02	-0.50 ± 0.03	-0.48 ± 0.03	-0.47 ± 0.03	-0.42 ± 0.03	-0.46 ± 0.03	-0.42 ± 0.03	-0.43 ± 0.03	-0.43 ± 0.03	-0.42 ± 0.02	-0.35 ± 0.03	-0.34 ± 0.03	-0.23 ± 0.04	<0.0001	0.014 ± 0.002
Non-worker	36,933	0.08 ± 0.02	0.14 ± 0.02	0.18 ± 0.02	0.13 ± 0.02	0.18 ± 0.02	0.15 ± 0.02	0.16 ± 0.02	0.20 ± 0.02	0.26 ± 0.02	0.18 ± 0.01	0.33 ± 0.02	0.31 ± 0.02	0.41 ± 0.02	<0.0001	0.018 ± 0.001
Weight status (*P* for interaction = 0.14)[d,e]																
Underweight	6743	0.05 ± 0.04	0.14 ± 0.04	0.12 ± 0.05	0.12 ± 0.04	0.11 ± 0.04	0.06 ± 0.04	0.12 ± 0.04	0.13 ± 0.04	0.08 ± 0.05	0.09 ± 0.02	0.16 ± 0.04	0.17 ± 0.05	0.29 ± 0.05	0.02	0.007 ± 0.003
Normal weight	59,611	-0.05 ± 0.01	-0.01 ± 0.02	0.02 ± 0.02	-0.01 ± 0.01	0.03 ± 0.02	-0.02 ± 0.01	0.01 ± 0.02	0.02 ± 0.02	0.04 ± 0.02	0.01 ± 0.01	0.10 ± 0.02	0.09 ± 0.02	0.20 ± 0.02	<0.0001	0.011 ± 0.001
Overweight	22,173	-0.17 ± 0.02	-0.19 ± 0.03	-0.12 ± 0.03	-0.18 ± 0.03	-0.12 ± 0.03	-0.14 ± 0.03	-0.13 ± 0.03	-0.10 ± 0.03	-0.05 ± 0.03	-0.13 ± 0.01	0.02 ± 0.03	0.01 ± 0.03	0.06 ± 0.03	<0.0001	0.015 ± 0.002
Current smoking (*P* for interaction = 0.87)[d]																
No	69,788	0.00 ± 0.01	0.04 ± 0.01	0.07 ± 0.01	0.04 ± 0.01	0.08 ± 0.01	0.03 ± 0.01	0.06 ± 0.01	0.08 ± 0.01	0.10 ± 0.01	0.07 ± 0.01	0.17 ± 0.01	0.17 ± 0.01	0.25 ± 0.01	<0.0001	0.012 ± 0.001
Yes	18,739	-0.37 ± 0.02	-0.38 ± 0.03	-0.29 ± 0.03	-0.34 ± 0.03	-0.31 ± 0.03	-0.33 ± 0.03	-0.29 ± 0.03	-0.30 ± 0.03	-0.28 ± 0.03	-0.32 ± 0.02	-0.27 ± 0.03	-0.27 ± 0.03	-0.13 ± 0.03	<0.0001	0.010 ± 0.002

[a] Values are mean ± standard error scores unless otherwise indicated. The dietary pattern score represents standardized variables with mean 0 and standard deviation 1. Negative scores indicate low adherence to the dietary pattern, whereas positive scores indicate high adherence. Adjustment was made for sex, age category, occupation, weight status, and current smoking. The number of participants in each category is shown in Table 1.

[b] A linear trend test was used with the survey year as a continuous variable in linear regression.

[c] *P* values for regression coefficients are theoretically identical to *P* values for trend.

[d] To calculate *P* values for interaction, the product term of the time and variable of stratification was added into the linear regression model.

[e] Defined based on body mass index (kg/m^2): <18.5 for underweight, ≥18.5 to <25 for normal weight, and ≥25 for overweight (including obese).

Table S7. Thirteen-year trends (2003-2015) in the "animal food and oil" dietary pattern score (factor 3): National Health and Nutrition Survey, Japan[a]

		Year													*P* for	Per-year change
	n	2003	2004	2005	2006	2007	2008	2009	2010	2011	2012	2013	2014	2015	trend[b]	β ± SE[c]
All	88,527	-0.07 ± 0.01	-0.11 ± 0.01	-0.06 ± 0.01	-0.02 ± 0.01	-0.01 ± 0.01	-0.09 ± 0.01	-0.01 ± 0.01	0.01 ± 0.01	-0.03 ± 0.01	0.03 ± 0.01	0.05 ± 0.01	0.11 ± 0.01	0.20 ± 0.01	<0.0001	0.017 ± 0.001
Sex (*P* for interaction = 0.77)[d]																
Male	39,557	0.19 ± 0.02	0.15 ± 0.02	0.21 ± 0.02	0.24 ± 0.02	0.28 ± 0.02	0.17 ± 0.02	0.26 ± 0.02	0.27 ± 0.02	0.25 ± 0.02	0.30 ± 0.01	0.37 ± 0.02	0.42 ± 0.02	0.50 ± 0.02	<0.0001	0.020 ± 0.001
Female	48,970	-0.28 ± 0.01	-0.32 ± 0.01	-0.28 ± 0.01	-0.23 ± 0.01	-0.25 ± 0.01	-0.29 ± 0.01	-0.23 ± 0.01	-0.20 ± 0.01	-0.25 ± 0.02	-0.20 ± 0.01	-0.21 ± 0.02	-0.13 ± 0.02	-0.05 ± 0.02	<0.0001	0.014 ± 0.001
Age category (*P* for interaction <0.0001)[d]																
20-34 years	10,983	0.37 ± 0.03	0.33 ± 0.03	0.34 ± 0.04	0.38 ± 0.03	0.33 ± 0.04	0.27 ± 0.04	0.29 ± 0.04	0.33 ± 0.04	0.31 ± 0.04	0.33 ± 0.02	0.28 ± 0.04	0.32 ± 0.04	0.46 ± 0.05	0.68	-0.001 ± 0.003
35-49 years	19,727	0.32 ± 0.02	0.25 ± 0.03	0.29 ± 0.03	0.33 ± 0.03	0.40 ± 0.02	0.19 ± 0.03	0.40 ± 0.03	0.34 ± 0.03	0.30 ± 0.03	0.34 ± 0.01	0.30 ± 0.03	0.44 ± 0.03	0.44 ± 0.03	<0.0001	0.008 ± 0.002
50-64 years	25,732	-0.05 ± 0.02	-0.05 ± 0.02	0.03 ± 0.02	0.06 ± 0.02	0.02 ± 0.02	0.01 ± 0.02	0.09 ± 0.02	0.07 ± 0.02	0.08 ± 0.02	0.13 ± 0.01	0.16 ± 0.02	0.20 ± 0.02	0.33 ± 0.03	<0.0001	0.023 ± 0.002
≥65 years	32,085	-0.50 ± 0.02	-0.53 ± 0.02	-0.48 ± 0.02	-0.44 ± 0.02	-0.42 ± 0.02	-0.46 ± 0.02	-0.46 ± 0.02	-0.36 ± 0.02	-0.44 ± 0.02	-0.35 ± 0.01	-0.29 ± 0.02	-0.25 ± 0.02	-0.16 ± 0.02	<0.0001	0.024 ± 0.001
Occupation (*P* for interaction = 0.01)[d]																
Professional/manager	13,082	0.27 ± 0.03	0.20 ± 0.03	0.27 ± 0.03	0.28 ± 0.03	0.27 ± 0.03	0.15 ± 0.03	0.35 ± 0.03	0.29 ± 0.03	0.30 ± 0.03	0.33 ± 0.02	0.34 ± 0.03	0.45 ± 0.04	0.44 ± 0.03	<0.0001	0.015 ± 0.002
Sales/service/clerical	21,555	0.12 ± 0.02	0.04 ± 0.02	0.12 ± 0.02	0.11 ± 0.02	0.14 ± 0.02	0.07 ± 0.02	0.14 ± 0.02	0.14 ± 0.02	0.13 ± 0.02	0.17 ± 0.01	0.14 ± 0.03	0.21 ± 0.03	0.36 ± 0.03	<0.0001	0.013 ± 0.002

Security/transportation/ labor	16,957	0.19 ± 0.03	0.16 ± 0.03	0.20 ± 0.03	0.27 ± 0.03	0.30 ± 0.03	0.20 ± 0.03	0.26 ± 0.03	0.29 ± 0.03	0.29 ± 0.03	0.30 ± 0.02	0.30 ± 0.03	0.37 ± 0.03	0.46 ± 0.04	<0.0001	0.016 ± 0.002
Non-worker	36,933	-0.42 ± 0.02	-0.42 ± 0.02	-0.41 ± 0.02	-0.34 ± 0.02	-0.35 ± 0.02	-0.40 ± 0.02	-0.36 ± 0.02	-0.31 ± 0.02	-0.38 ± 0.02	-0.29 ± 0.01	-0.22 ± 0.02	-0.19 ± 0.02	-0.11 ± 0.02	<0.0001	0.021 ± 0.001
Weight status (*P* for interaction = 0.05)[d,e]																
Underweight	6743	-0.26 ± 0.04	-0.32 ± 0.04	-0.19 ± 0.04	-0.16 ± 0.04	-0.16 ± 0.04	-0.24 ± 0.04	-0.18 ± 0.04	-0.24 ± 0.04	-0.17 ± 0.04	-0.17 ± 0.02	-0.14 ± 0.04	-0.13 ± 0.04	0.01 ± 0.04	<0.0001	0.014 ± 0.003
Normal weight	59,611	-0.09 ± 0.01	-0.10 ± 0.01	-0.06 ± 0.01	-0.02 ± 0.01	-0.02 ± 0.01	-0.09 ± 0.01	-0.02 ± 0.01	0.02 ± 0.01	-0.04 ± 0.02	0.03 ± 0.01	0.04 ± 0.01	0.11 ± 0.01	0.20 ± 0.02	<0.0001	0.017 ± 0.001
Overweight	22,173	0.02 ± 0.02	-0.06 ± 0.03	-0.01 ± 0.03	0.02 ± 0.02	0.05 ± 0.02	-0.04 ± 0.02	0.05 ± 0.02	0.06 ± 0.03	0.04 ± 0.03	0.09 ± 0.01	0.13 ± 0.03	0.19 ± 0.03	0.25 ± 0.03	<0.0001	0.018 ± 0.002
Current smoking (*P* for interaction = 0.03)[d]																
No	69,788	-0.18 ± 0.01	-0.22 ± 0.01	-0.16 ± 0.01	-0.12 ± 0.01	-0.13 ± 0.01	-0.17 ± 0.01	-0.11 ± 0.01	-0.08 ± 0.01	-0.15 ± 0.01	-0.07 ± 0.01	-0.05 ± 0.01	0.02 ± 0.01	0.09 ± 0.01	<0.0001	0.018 ± 0.001
Yes	18,739	0.31 ± 0.02	0.31 ± 0.03	0.32 ± 0.03	0.35 ± 0.03	0.40 ± 0.03	0.22 ± 0.03	0.37 ± 0.03	0.33 ± 0.03	0.44 ± 0.03	0.40 ± 0.02	0.42 ± 0.03	0.46 ± 0.03	0.62 ± 0.04	<0.0001	0.015 ± 0.002

[a] Values are mean ± standard error scores unless otherwise indicated. The dietary pattern score represents standardized variables with mean 0 and standard deviation 1. Negative scores indicate low adherence to the dietary pattern, whereas positive scores indicate high adherence. Adjustment was made for sex, age category, occupation, weight status, and current smoking. The number of participants in each category is shown in Table 1.

[b] A linear trend test was used with the survey year as a continuous variable in linear regression.

[c] *P* values for regression coefficients are theoretically identical to *P* values for trend.

[d] To calculate *P* values for interaction, the product term of the time and variable of stratification was added into the linear regression model.

[e] Defined based on body mass index (kg/m^2): <18.5 for underweight, ≥18.5 to <25 for normal weight, and ≥25 for overweight (including obese).

Table S8. Factor loadings for dietary patterns identified among the 68,810 participants of the National Health and Nutrition Survey, Japan 2003-2011 and 2013-2015 (excluding 2012 data)[a]

	Factor 1<br>"Plant food and fish" pattern	Factor 2<br>"Bread and dairy" pattern	Factor 3<br>"Animal food and oil" pattern
Rice	**0.34**	**-0.55**	0.14
Bread	-0.19	**0.63**	0.17
Noodles	-0.21	0.04	0.02
Other grains	0.01	0.05	0.25
Potatoes	**0.36**	-0.02	0.17
Sugar	**0.33**	**0.33**	0.10
Pulses	**0.41**	-0.04	-0.06
Nuts	0.19	0.17	-0.03
Green and yellow vegetables	**0.51**	0.18	0.07
Other vegetables	**0.48**	-0.01	**0.34**
Vegetable and fruit juice	-0.03	0.14	0.04
Pickled vegetables	0.29	-0.14	-0.09
Fruit	**0.43**	**0.40**	-0.21
Mushrooms	**0.30**	0.04	0.08
Seaweeds	**0.32**	-0.02	-0.04
Fish	**0.30**	-0.04	-0.16
Shellfish	0.08	-0.06	0.16
Sea products	0.27	-0.12	-0.03
Red meat	0.01	-0.11	**0.48**
Processed meat	-0.10	0.13	**0.37**
Chicken	0.00	-0.05	0.24
Eggs	0.12	-0.03	**0.40**
Dairy products	0.15	**0.55**	-0.06
Animal fat	-0.09	0.28	0.25
Vegetable oil	0.00	0.10	**0.64**
Confectioneries	-0.01	0.23	-0.08
Alcoholic beverages	-0.04	-0.24	0.28
Tea	**0.34**	0.06	-0.21
Coffee	-0.13	0.21	0.29
Soft drinks	-0.13	0.00	0.20
Salt-based seasonings	**0.60**	-0.24	0.16
Variability explained (%)	7.49	5.62	5.60

[a] Dietary patterns were identified using principal component analysis based on intakes of the 31 food groups (g/day). Absolute factor loading values ≥0.30 are presented in bold. Total variability explained was 18.70%.

Table S9. Secular trends in the "plant food and fish" dietary pattern score (factor 1): National Health and Nutrition Survey, Japan 2003-2011 and 2013-2015 (excluding 2012 data)[a]

		Year												*P* for	Per-year change
	n	2003	2004	2005	2006	2007	2008	2009	2010	2011	2013	2014	2015	trend [b]	β ± SE [c]
All	68,810	0.22 ± 0.01	0.11 ± 0.01	0.18 ± 0.01	0.14 ± 0.01	0.04 ± 0.01	0.01 ± 0.01	-0.02 ± 0.01	-0.08 ± 0.01	-0.13 ± 0.01	-0.18 ± 0.01	-0.20 ± 0.01	-0.22 ± 0.01	<0.0001	-0.038 ± 0.001
Sex (*P* for interaction = 0.71)[d]															
Male	30,845	0.32 ± 0.02	0.18 ± 0.02	0.26 ± 0.02	0.23 ± 0.02	0.12 ± 0.02	0.10 ± 0.02	0.05 ± 0.02	0.00 ± 0.02	-0.07 ± 0.02	-0.09 ± 0.02	-0.12 ± 0.02	-0.16 ± 0.02	<0.0001	-0.040 ± 0.001
Female	37,965	0.14 ± 0.01	0.05 ± 0.02	0.12 ± 0.02	0.07 ± 0.02	-0.02 ± 0.02	-0.07 ± 0.02	-0.08 ± 0.02	-0.15 ± 0.02	-0.18 ± 0.02	-0.25 ± 0.02	-0.27 ± 0.02	-0.26 ± 0.02	<0.0001	-0.037 ± 0.001
Age category (*P* for interaction = 0.06)[d]															
20-34 years	8872	-0.33 ± 0.02	-0.36 ± 0.03	-0.35 ± 0.03	-0.42 ± 0.03	-0.46 ± 0.03	-0.50 ± 0.03	-0.48 ± 0.03	-0.53 ± 0.03	-0.62 ± 0.03	-0.59 ± 0.03	-0.65 ± 0.04	-0.61 ± 0.04	<0.0001	-0.028 ± 0.002
35-49 years	15,386	-0.16 ± 0.02	-0.31 ± 0.02	-0.23 ± 0.02	-0.25 ± 0.02	-0.41 ± 0.02	-0.42 ± 0.02	-0.41 ± 0.02	-0.51 ± 0.02	-0.42 ± 0.02	-0.58 ± 0.02	-0.56 ± 0.02	-0.57 ± 0.02	<0.0001	-0.035 ± 0.002
50-64 years	20,047	0.39 ± 0.02	0.28 ± 0.02	0.34 ± 0.02	0.26 ± 0.02	0.20 ± 0.02	0.15 ± 0.02	0.08 ± 0.02	0.03 ± 0.02	-0.05 ± 0.02	-0.13 ± 0.03	-0.13 ± 0.03	-0.22 ± 0.03	<0.0001	-0.050 ± 0.002
≥65 years	24,505	0.53 ± 0.02	0.38 ± 0.02	0.50 ± 0.02	0.50 ± 0.02	0.37 ± 0.02	0.34 ± 0.02	0.32 ± 0.02	0.25 ± 0.02	0.22 ± 0.02	0.19 ± 0.02	0.14 ± 0.02	0.15 ± 0.02	<0.0001	-0.033 ± 0.002
Occupation (*P* for interaction = 0.66)[d]															
Professional/manager	10,337	0.12 ± 0.03	0.01 ± 0.03	0.08 ± 0.03	0.03 ± 0.03	-0.09 ± 0.03	-0.14 ± 0.03	-0.09 ± 0.03	-0.14 ± 0.03	-0.23 ± 0.03	-0.30 ± 0.03	-0.26 ± 0.03	-0.29 ± 0.03	<0.0001	-0.035 ± 0.002
Sales/service/clerical	16,717	-0.01 ± 0.02	-0.09 ± 0.02	-0.07 ± 0.02	-0.08 ± 0.02	-0.18 ± 0.02	-0.20 ± 0.02	-0.25 ± 0.02	-0.30 ± 0.02	-0.38 ± 0.02	-0.41 ± 0.02	-0.40 ± 0.02	-0.43 ± 0.03	<0.0001	-0.038 ± 0.002

Security/transportation/labor	13,113	0.37 ± 0.02	0.23 ± 0.03	0.28 ± 0.03	0.26 ± 0.03	0.1153 ± 0.03	0.16 ± 0.03	0.10 ± 0.03	-0.01 ± 0.03	-0.03 ± 0.03	-0.12 ± 0.03	-0.14 ± 0.03	-0.23 ± 0.04	<0.0001	-0.047 ± 0.002
Non-worker	28,643	0.32 ± 0.02	0.20 ± 0.02	0.32 ± 0.02	0.26 ± 0.02	0.17 ± 0.02	0.11 ± 0.02	0.09 ± 0.02	0.03 ± 0.02	0.00 ± 0.02	-0.02 ± 0.02	-0.09 ± 0.02	-0.06 ± 0.02	<0.0001	-0.035 ± 0.001
Weight status (*P* for interaction <0.0001) [d, e]															
Underweight	5245	-0.07 ± 0.04	-0.21 ± 0.04	-0.08 ± 0.04	-0.05 ± 0.04	-0.18 ± 0.04	-0.24 ± 0.04	-0.26 ± 0.04	-0.30 ± 0.04	-0.33 ± 0.04	-0.39 ± 0.04	-0.35 ± 0.04	-0.39 ± 0.04	<0.0001	-0.028 ± 0.003
Normal weight	46,361	0.22 ± 0.01	0.11 ± 0.01	0.19 ± 0.01	0.14 ± 0.01	0.02 ± 0.01	0.01 ± 0.01	-0.02 ± 0.01	-0.08 ± 0.01	-0.14 ± 0.02	-0.18 ± 0.02	-0.20 ± 0.02	-0.22 ± 0.02	<0.0001	-0.038 ± 0.001
Overweight	17,204	0.33 ± 0.02	0.20 ± 0.03	0.24 ± 0.03	0.21 ± 0.02	0.15 ± 0.03	0.09 ± 0.02	0.06 ± 0.02	-0.04 ± 0.03	-0.05 ± 0.03	-0.10 ± 0.03	-0.16 ± 0.03	-0.16 ± 0.03	<0.0001	-0.041 ± 0.002
Current smoking (*P* for interaction = 0.98)[d]															
No	53,653	0.31 ± 0.01	0.17 ± 0.01	0.26 ± 0.01	0.21 ± 0.01	0.10 ± 0.01	0.08 ± 0.01	0.06 ± 0.01	-0.01 ± 0.01	-0.06 ± 0.01	-0.09 ± 0.01	-0.13 ± 0.01	-0.14 ± 0.01	<0.0001	-0.038 ± 0.001
Yes	15,157	-0.08 ± 0.02	-0.13 ± 0.02	-0.11 ± 0.03	-0.09 ± 0.02	-0.19 ± 0.02	-0.24 ± 0.02	-0.28 ± 0.02	-0.33 ± 0.03	-0.40 ± 0.03	-0.49 ± 0.03	-0.44 ± 0.03	-0.49 ± 0.03	<0.0001	-0.038 ± 0.002

[a] Values are mean ± standard error scores unless otherwise indicated. The dietary pattern score represents standardized variables with mean 0 and standard deviation 1. Negative scores indicate low adherence to the dietary pattern, whereas positive scores indicate high adherence. Adjustment was made for sex, age category, occupation, weight status, and current smoking. The number of participants in each category is shown in Table 1.

[b] A linear trend test was used with the survey year as a continuous variable in linear regression.

[c] *P* values for regression coefficients are theoretically identical to *P* values for trend.

[d] To calculate *P* values for interaction, the product term of the time and variable of stratification was added into the linear regression model.

[e] Defined based on body mass index (kg/m^2): <18.5 for underweight, ≥18.5 to <25 for normal weight, and ≥25 for overweight (including obese).

Table S10. Secular trends in the "bread and dairy" dietary pattern score (factor 2): National Health and Nutrition Survey, Japan 2003-2011 and 2013-2015 (excluding 2012 data)[a]

		Year												*P* for	Per-year change
	n	2003	2004	2005	2006	2007	2008	2009	2010	2011	2013	2014	2015	trend [b]	β ± SE [c]
All	68,810	-0.08 ± 0.01	-0.05 ± 0.01	-0.01 ± 0.01	-0.05 ± 0.01	0.00 ± 0.01	-0.05 ± 0.01	-0.02 ± 0.01	-0.01 ± 0.01	0.02 ± 0.01	0.08 ± 0.01	0.07 ± 0.01	0.17 ± 0.01	<0.0001	0.015 ± 0.001
Sex (*P* for interaction = 0.002)[d]															
Male	30,845	-0.35 ± 0.02	-0.32 ± 0.02	-0.30 ± 0.02	-0.30 ± 0.02	-0.27 ± 0.02	-0.31 ± 0.02	-0.29 ± 0.02	-0.28 ± 0.02	-0.27 ± 0.02	-0.20 ± 0.02	-0.20 ± 0.02	-0.10 ± 0.02	<0.0001	0.015 ± 0.002
Female	37,965	0.14 ± 0.01	0.17 ± 0.02	0.22 ± 0.02	0.16 ± 0.02	0.21 ± 0.02	0.16 ± 0.02	0.20 ± 0.02	0.21 ± 0.02	0.25 ± 0.02	0.30 ± 0.02	0.29 ± 0.02	0.38 ± 0.02	<0.0001	0.015 ± 0.001
Age category (*P* for interaction <0.0001)[d]															
20-34 years	8872	-0.16 ± 0.03	-0.17 ± 0.03	-0.13 ± 0.03	-0.07 ± 0.03	-0.14 ± 0.03	-0.19 ± 0.03	-0.17 ± 0.03	-0.15 ± 0.04	-0.12 ± 0.04	-0.18 ± 0.04	-0.09 ± 0.04	-0.13 ± 0.04	0.68	0.001 ± 0.003
35-49 years	15,386	-0.14 ± 0.02	-0.11 ± 0.03	-0.10 ± 0.03	-0.11 ± 0.03	-0.10 ± 0.02	-0.14 ± 0.03	-0.08 ± 0.02	-0.07 ± 0.03	-0.09 ± 0.03	-0.09 ± 0.03	-0.08 ± 0.03	-0.03 ± 0.03	0.002	0.006 ± 0.002
50-64 years	20,047	-0.03 ± 0.02	-0.04 ± 0.02	0.06 ± 0.02	-0.03 ± 0.02	0.08 ± 0.02	-0.01 ± 0.02	0.03 ± 0.02	-0.01 ± 0.02	0.04 ± 0.03	0.09 ± 0.03	0.09 ± 0.03	0.21 ± 0.03	<0.0001	0.013 ± 0.002
≥65 years	24,505	-0.07 ± 0.02	0.02 ± 0.02	0.03 ± 0.02	-0.02 ± 0.02	0.03 ± 0.02	0.02 ± 0.02	0.03 ± 0.02	0.09 ± 0.02	0.12 ± 0.02	0.24 ± 0.02	0.19 ± 0.02	0.33 ± 0.02	<0.0001	0.027 ± 0.002
Occupation (*P* for interaction <0.0001)[d]															
Professional/manager	10,337	-0.04 ± 0.03	-0.03 ± 0.03	-0.02 ± 0.03	-0.08 ± 0.03	0.00 ± 0.03	-0.08 ± 0.03	-0.03 ± 0.03	-0.02 ± 0.03	-0.04 ± 0.03	-0.02 ± 0.03	0.01 ± 0.04	0.03 ± 0.03	0.13	0.004 ± 0.003
Sales/service/clerical	16,717	-0.04 ± 0.02	-0.04 ± 0.03	0.03 ± 0.03	0.01 ± 0.02	0.01 ± 0.02	-0.05 ± 0.03	0.00 ± 0.02	-0.02 ± 0.03	-0.02 ± 0.03	0.02 ± 0.03	0.02 ± 0.03	0.14 ± 0.03	0.0001	0.007 ± 0.002

Security/transportation/labor	13,113	-0.51 ± 0.02	-0.51 ± 0.03	-0.49 ± 0.03	-0.48 ± 0.03	-0.43 ± 0.03	-0.47 ± 0.03	-0.43 ± 0.03	-0.44 ± 0.03	-0.43 ± 0.03	-0.36 ± 0.03	-0.35 ± 0.03	-0.24 ± 0.04	<0.0001	0.017 ± 0.002
Non-worker	28,643	0.08 ± 0.02	0.14 ± 0.02	0.18 ± 0.02	0.13 ± 0.02	0.18 ± 0.02	0.15 ± 0.02	0.16 ± 0.02	0.20 ± 0.02	0.27 ± 0.02	0.33 ± 0.02	0.31 ± 0.02	0.41 ± 0.02	<0.0001	0.022 ± 0.001
Weight status (*P* for interaction = 0.05)[d,e]															
Underweight	5245	0.06 ± 0.04	0.14 ± 0.04	0.12 ± 0.05	0.12 ± 0.05	0.11 ± 0.04	0.06 ± 0.04	0.13 ± 0.04	0.14 ± 0.04	0.09 ± 0.05	0.16 ± 0.04	0.17 ± 0.05	0.29 ± 0.05	0.002	0.010 ± 0.003
Normal weight	46,361	-0.06 ± 0.01	-0.02 ± 0.02	0.02 ± 0.02	-0.01 ± 0.01	0.03 ± 0.02	-0.03 ± 0.01	0.01 ± 0.01	0.01 ± 0.02	0.04 ± 0.02	0.09 ± 0.02	0.08 ± 0.02	0.19 ± 0.02	<0.0001	0.014 ± 0.001
Overweight	17,204	-0.18 ± 0.02	-0.20 ± 0.03	-0.13 ± 0.03	-0.19 ± 0.03	-0.12 ± 0.03	-0.15 ± 0.03	-0.13 ± 0.03	-0.11 ± 0.03	-0.06 ± 0.03	0.01 ± 0.03	0.01 ± 0.03	0.06 ± 0.03	<0.0001	0.019 ± 0.002
Current smoking (*P* for interaction = 0.85)[d]															
No	53,653	0.00 ± 0.01	0.05 ± 0.01	0.07 ± 0.01	0.04 ± 0.01	0.08 ± 0.01	0.03 ± 0.01	0.06 ± 0.01	0.08 ± 0.01	0.10 ± 0.01	0.17 ± 0.01	0.17 ± 0.01	0.25 ± 0.01	<0.0001	0.016 ± 0.001
Yes	15,157	-0.38 ± 0.02	-0.39 ± 0.03	-0.31 ± 0.03	-0.35 ± 0.03	-0.32 ± 0.03	-0.34 ± 0.03	-0.30 ± 0.03	-0.30 ± 0.03	-0.28 ± 0.03	-0.28 ± 0.03	-0.28 ± 0.03	-0.14 ± 0.03	<0.0001	0.013 ± 0.002

[a] Values are mean ± standard error scores unless otherwise indicated. The dietary pattern score represents standardized variables with mean 0 and standard deviation 1. Negative scores indicate low adherence to the dietary pattern, whereas positive scores indicate high adherence. Adjustment was made for sex, age category, occupation, weight status, and current smoking. The number of participants in each category is shown in Table 1.

[b] A linear trend test was used with the survey year as a continuous variable in linear regression.

[c] *P* values for regression coefficients are theoretically identical to *P* values for trend.

[d] To calculate *P* values for interaction, the product term of the time and variable of stratification was added into the linear regression model.

[e] Defined based on body mass index (kg/m^2): <18.5 for underweight, ≥18.5 to <25 for normal weight, and ≥25 for overweight (including obese).

Table S11. Secular trends in the "animal food and oil" dietary pattern score (factor 3): National Health and Nutrition Survey, Japan 2003-2011 and 2013-2015 (excluding 2012 data)[1]

	n	Year												*P* for trend [b]	Per-year change β ± SE [c]
		2003	2004	2005	2006	2007	2008	2009	2010	2011	2013	2014	2015		
All	68,810	-0.06 ± 0.01	-0.10 ± 0.01	-0.05 ± 0.01	-0.02 ± 0.01	-0.01 ± 0.01	-0.08 ± 0.01	0.00 ± 0.01	0.01 ± 0.01	-0.02 ± 0.01	0.06 ± 0.01	0.12 ± 0.01	0.20 ± 0.01	<0.0001	0.018 ± 0.001
Sex (*P* for interaction = 0.45)[d]															
Male	30,845	0.19 ± 0.02	0.15 ± 0.02	0.21 ± 0.02	0.24 ± 0.02	0.28 ± 0.02	0.17 ± 0.02	0.26 ± 0.02	0.27 ± 0.02	0.25 ± 0.02	0.38 ± 0.02	0.42 ± 0.02	0.50 ± 0.02	<0.0001	0.022 ± 0.002
Female	37,965	-0.27 ± 0.01	-0.30 ± 0.01	-0.26 ± 0.01	-0.22 ± 0.01	-0.24 ± 0.01	-0.28 ± 0.01	-0.22 ± 0.01	-0.19 ± 0.01	-0.24 ± 0.02	-0.20 ± 0.02	-0.12 ± 0.02	-0.04 ± 0.02	<0.0001	0.015 ± 0.001
Age category (*P* for interaction <0.0001)[d]															
20-34 years	8872	0.36 ± 0.03	0.32 ± 0.03	0.33 ± 0.04	0.37 ± 0.03	0.33 ± 0.04	0.26 ± 0.04	0.28 ± 0.04	0.32 ± 0.04	0.30 ± 0.04	0.27 ± 0.04	0.32 ± 0.04	0.45 ± 0.05	0.72	-0.001 ± 0.003
35-49 years	15,386	0.32 ± 0.02	0.25 ± 0.03	0.29 ± 0.03	0.32 ± 0.03	0.39 ± 0.03	0.19 ± 0.03	0.40 ± 0.03	0.34 ± 0.03	0.30 ± 0.03	0.30 ± 0.03	0.44 ± 0.03	0.43 ± 0.03	<0.0001	0.009 ± 0.002
50-64 years	20,047	-0.04 ± 0.02	-0.04 ± 0.02	0.04 ± 0.02	0.06 ± 0.02	0.03 ± 0.02	0.01 ± 0.02	0.10 ± 0.02	0.07 ± 0.02	0.09 ± 0.02	0.17 ± 0.02	0.20 ± 0.02	0.33 ± 0.03	<0.0001	0.024 ± 0.002
≥65 years	24,505	-0.49 ± 0.02	-0.52 ± 0.02	-0.47 ± 0.02	-0.43 ± 0.02	-0.41 ± 0.02	-0.45 ± 0.02	-0.45 ± 0.02	-0.35 ± 0.02	-0.43 ± 0.02	-0.27 ± 0.02	-0.24 ± 0.02	-0.15 ± 0.02	<0.0001	0.026 ± 0.001
Occupation (*P* for interaction = 0.01)[d]															
Professional/manager	10,337	0.27 ± 0.03	0.20 ± 0.03	0.27 ± 0.03	0.28 ± 0.03	0.27 ± 0.03	0.15 ± 0.03	0.35 ± 0.03	0.29 ± 0.03	0.30 ± 0.03	0.34 ± 0.03	0.45 ± 0.04	0.44 ± 0.03	<0.0001	0.016 ± 0.003
Sales/service/clerical	16,717	0.12 ± 0.02	0.04 ± 0.02	0.13 ± 0.02	0.12 ± 0.02	0.14 ± 0.02	0.08 ± 0.02	0.15 ± 0.02	0.15 ± 0.02	0.13 ± 0.03	0.15 ± 0.03	0.22 ± 0.03	0.37 ± 0.03	<0.0001	0.014 ± 0.002

Security/transportation/labor	13,113	0.19 ± 0.03	0.16 ± 0.03	0.21 ± 0.03	0.27 ± 0.03	0.30 ± 0.03	0.21 ± 0.03	0.26 ± 0.03	0.30 ± 0.03	0.29 ± 0.03	0.30 ± 0.03	0.37 ± 0.03	0.46 ± 0.04	<0.0001	0.017 ± 0.002
Non-worker	28,643	-0.41 ± 0.02	-0.41 ± 0.02	-0.39 ± 0.02	-0.33 ± 0.02	-0.34 ± 0.02	-0.39 ± 0.02	-0.34 ± 0.02	-0.29 ± 0.02	-0.36 ± 0.02	-0.21 ± 0.02	-0.17 ± 0.02	-0.10 ± 0.02	<0.0001	0.022 ± 0.001
Weight status (*P* for interaction = 0.05) [d, e]															
Underweight	5245	-0.25 ± 0.04	-0.32 ± 0.04	-0.16 ± 0.04	-0.16 ± 0.04	-0.16 ± 0.04	-0.24 ± 0.04	-0.18 ± 0.04	-0.23 ± 0.04	-0.16 ± 0.04	-0.14 ± 0.04	-0.13 ± 0.04	0.02 ± 0.05	<0.0001	0.015 ± 0.003
Normal weight	46,361	-0.08 ± 0.01	-0.09 ± 0.01	-0.05 ± 0.01	-0.01 ± 0.01	-0.01 ± 0.01	-0.08 ± 0.01	-0.01 ± 0.01	0.03 ± 0.01	-0.03 ± 0.02	0.05 ± 0.01	0.12 ± 0.01	0.20 ± 0.02	<0.0001	0.018 ± 0.001
Overweight	17,204	0.03 ± 0.02	-0.06 ± 0.03	-0.01 ± 0.03	0.03 ± 0.02	0.05 ± 0.02	-0.03 ± 0.02	0.05 ± 0.02	0.06 ± 0.03	0.04 ± 0.03	0.14 ± 0.03	0.20 ± 0.03	0.26 ± 0.03	<0.0001	0.019 ± 0.002
Current smoking (*P* for interaction = 0.04)[d]															
No	53,653	-0.17 ± 0.01	-0.22 ± 0.01	-0.15 ± 0.01	-0.12 ± 0.01	-0.12 ± 0.01	-0.16 ± 0.01	-0.11 ± 0.01	-0.08 ± 0.01	-0.14 ± 0.01	-0.04 ± 0.01	0.02 ± 0.01	0.09 ± 0.01	<0.0001	0.019 ± 0.001
Yes	15,157	0.31 ± 0.02	0.30 ± 0.03	0.31 ± 0.03	0.34 ± 0.03	0.40 ± 0.03	0.22 ± 0.03	0.36 ± 0.03	0.32 ± 0.03	0.43 ± 0.03	0.41 ± 0.03	0.46 ± 0.03	0.61 ± 0.04	<0.0001	0.017 ± 0.002

[a] Values are mean ± standard error scores unless otherwise indicated. The dietary pattern score represents standardized variables with mean 0 and standard deviation 1. Negative scores indicate low adherence to the dietary pattern, whereas positive scores indicate high adherence. Adjustment was made for sex, age category, occupation, weight status, and current smoking. The number of participants in each category is shown in Table 1.

[b] A linear trend test was used with the survey year as a continuous variable in linear regression.

[c] *P* values for regression coefficients are theoretically identical to *P* values for trend.

[d] To calculate *P* values for interaction, the product term of the time and variable of stratification was added into the linear regression model.

[e] Defined based on body mass index (kg/m^2): <18.5 for underweight, ≥18.5 to <25 for normal weight, and ≥25 for overweight (including obese).
